# Outer membrane protein 25 of *Brucella* suppresses TLR-mediated expression of proinflammatory cytokines through degradation of TLRs and adaptor proteins

**DOI:** 10.1016/j.jbc.2023.105309

**Published:** 2023-09-29

**Authors:** Subathra Murugan, Binita Roy Nandi, Varadendra Mazumdar, Kiranmai Joshi, Prachita Nandini, Swapna Namani, Padmaja Jakka, Girish K. Radhakrishnan

**Affiliations:** 1 Laboratory of Immunology and Microbial Pathogenesis, National Institute of Animal Biotechnology, Hyderabad, Telangana, India; 2 Centre for Biotechnology, Institute of Science and Technology, Jawaharlal Nehru Technological University, Hyderabad, India; 3 Regional Centre for Biotechnology (RCB), Faridabad, India

**Keywords:** Toll-like receptors, innate immunity, proinflammatory cytokines, ubiquitination, *Brucella*, outer membrane proteins

## Abstract

Toll-like receptors (TLRs) are essential components of innate immunity that serves as the first line of defense against the invaded microorganisms. However, successful infectious pathogens subvert TLR signaling to suppress the activation of innate and adaptive responses. *Brucella* species are infectious intracellular bacterial pathogens causing the worldwide zoonotic disease, brucellosis, that impacts economic growth of many countries. *Brucella* species are considered as stealthy bacterial pathogens as they efficiently evade or suppress host innate and adaptive immune responses for their chronic persistence. However, the bacterial effectors and their host targets for modulating the immune responses remain obscure. *Brucella* encodes various outer membrane proteins (Omps) that facilitate their invasion, intracellular replication, and immunomodulation. Outer membrane protein 25 (Omp25) of *Brucella* plays an important role in the immune modulation through suppression of proinflammatory cytokines. However, the mechanism and the signaling pathways that are targeted by Omp25 to attenuate the production of proinflammatory cytokines remain obscure. Here, we report that Omp25 and its variants, *viz*. Omp25b, Omp25c, and Omp25d, suppress production of proinflammatory cytokines that are mediated by various TLRs. Furthermore, we demonstrate that Omp25 and its variants promote enhanced ubiquitination and degradation of TLRs and their adaptor proteins to attenuate the expression of proinflammatory cytokines. Targeting multiple TLRs and adaptor proteins enables Omp25 to effectively suppress the expression of proinflammatory cytokines that are induced by diverse pathogen-associated molecular patterns. This can contribute to the defective adaptive immune response and the chronic persistence of *Brucella* in the host.

Innate immunity is the first line of defense against the invaded microorganisms where the Toll/interleukin-like receptors (TLRs) play a crucial role in the recognition of pathogen–associated molecular patterns (PAMPs) and driving various antimicrobial responses, including secretion of proinflammatory cytokines (1, 2). Human and mouse are reported to harbor 10 and 12 TLRs, respectively (3). Some of the TLRs, such as TLR2, TLR4, and TLR6, are located on the surface of the immune cells, whereas TLR9 and TLR3 are located on the endosomes inside the cells (4). TLRs constitute a leucine-rich domain at the cell surface that recognizes PAMPs and a TIR domain on the cytoplasmic side (5). There are five TIR domain–containing adaptor proteins, such as TIRAP (Toll/interleukin-1 receptor domain–containing adapter protein), MYD88 (myeloid differentiation primary response protein), TRIF (TIR domain–containing adapter-inducing interferon-β), TRAM (TRIF-related adaptor molecule), and SARM (sterile-alpha and armadillo motif–containing protein), which are selectively recruited to the TIR domain of respective TLRs upon ligand binding (6). The adaptor protein, MYD88, is shared by all the TLRs except for TLR3. TIRAP plays an essential role in TLR2 and TLR4-mediated signaling by bridging MYD88 to the TIR domain of plasma membrane–localized TLR2 and TLR4 (7). The adaptors, TRIF and TRAM, are required for MYD88-independent signaling from TLR4, which mainly occurs on endosomes that harbor internalized TLR4 (8). In addition to TLR4, TRIF serves as an adaptor for TLR3 signaling also (9). SARM is the TIR domain–containing adaptor protein that negatively regulates TLR3 and TKR4 signaling by interacting with TRIF and MYD88 (10, 11). Activation of TLRs by the respective ligands leads to recruitment of specific adaptor proteins, which acts as a platform for the assembly and activation of various downstream molecules that ultimately results in the activation of various transcription factors, such as NF-κB, AP-1, and IRF-3 (1, 9). Subsequently, these transcription factors translocate into the nucleus and drive the expression of various genes coding for proinflammatory cytokines (tumor necrosis factor alpha [TNF-α], interleukin 6 [IL6], and IL-1β), type I interferons (IFN-α and β), chemokines (CXCL8 [C-X-C motif chemokine ligand 8] and CXCL10 [C-X-C motif chemokine ligand 10]), and antimicrobial peptides (12, 13, 14). These cytokines and chemokines play a major role in stimulating and reshaping the adaptive immunity through activation and maturation of various antigen-presenting cells (15, 16, 17).

Many microbial pathogens subvert TLR-mediated signaling to suppress the innate and adaptive immune responses to successfully infect the host (18, 19, 20). The effector proteins of many bacterial pathogens target key proteins in the TLR signaling pathway such as TIR domain–containing adaptor proteins, kinases, and transcription factors (20). The TIR domain–containing effector proteins of *Escherichia coli* strain CFT073 (TcpC [Toll/interleukin-1 receptor domain–containing protein C]) and *Yersinia pestis* (YPTIR [Yersinia pestis TIR-domain protein]) are reported to target MYD88 to attenuate TLR signaling (21). The acetyl transferases encoded by *Vibrio parahaemolyticus*, *Salmonella typhimurium*, and *Yersinia* spp. mainly target mitogen-activated protein kinase kinase to subvert TLR signaling (22, 23, 24). Proteases encoded by *Bacillus anthracis*, *E. coli* (enteropathogenic *E. coli*/enterohemorrhagic *E. coli*), and *Chlamydia* spp. are reported to cleave various proteins of TLR signaling pathway, including mitogen-activated protein kinase kinase, c-Jun N-terminal kinase, and NF-κB (25, 26, 27).

*Brucella* species are infectious intracellular bacterial pathogens causing the zoonotic disease, brucellosis (28). Brucellosis poses a serious veterinary and public health problem with major impact on the economic growth of various countries. Moreover, the highly infectious species, such as *Brucella melitensis*, *Brucella abortus*, and *Brucella suis*, have been considered as the potential bioweapon agents or bioterrorism agents (29). There is no human vaccine available for brucellosis, and the antibiotic treatment of brucellosis remains inefficient because of prolonged duration of treatment and frequent occurrence of relapse. *Brucella* are considered as a stealthy bacterial pathogen as they efficiently evade or suppress the host innate and adaptive immune responses leading to a chronic infection in the host (30, 31, 32). However, the strategies employed by *Brucella* to subvert the host immune responses remain obscure. *Brucella* species encode the TIR domain–containing protein, TcpB, that efficiently suppresses TLR2- and TLR4-mediated NF-κB activation and production of proinflammatory cytokines (18, 33, 34, 35). Studies have demonstrated that TcpB targets the TLR2/4 adaptor protein, TIRAP, where it induces targeted ubiquitination and degradation of TIRAP (34, 36). TcpB has been reported to interact with the host protein, CLIP170, which harbors a putative ubiquitin ligase property (37). TcpB also attenuated canonical and noncanonical inflammasome signaling by inducing degradation of key proteins in these signaling pathways (35).

*Brucella* spp. encodes another effector protein, outer membrane protein 25 (Omp25), which has been reported to suppress NF-κB activation and production of proinflammatory cytokines in macrophages (38, 39, 40). Omp25 belongs to group III Omps that constitute the Omp25 and Omp31 families (41, 42). There are three paralogs reported in the Omp25 family, such as Omp25b, Omp25c, and Omp25d (41). Omp25 suppresses secretion of TNF-α, IL-6, IL-1β, and IL-12 by mouse and human macrophages (39, 40, 43, 44). Omp25 has been reported to modulate miRNA expression in the *Brucella*-infected cells to attenuate production of proinflammatory cytokines (40). However, the signaling pathways and the proteins, which are targeted by Omp25 to attenuate NF-κB activation and secretion of proinflammatory cytokines, remain obscure. Here, we investigated the effect of Omp25 and its paralogs on TLR signaling pathways. We found that Omp25 and its variants negatively regulate NF-κB activation and production of proinflammatory cytokines mediated by various TLRs. Furthermore, we found that Omp25 promotes enhanced ubiquitination and degradation of key proteins in the TLR signaling pathways to inhibit the signaling cascade. Our studies revealed the novel property of Omp25 to negatively regulate TLR signaling that enables *Brucella* to subvert host innate and adaptive immune responses for their chronic persistence.

## Results

### Omp25 and its variants suppress TLR-mediated expression of proinflammatory cytokines in macrophages

**Figure 1:**
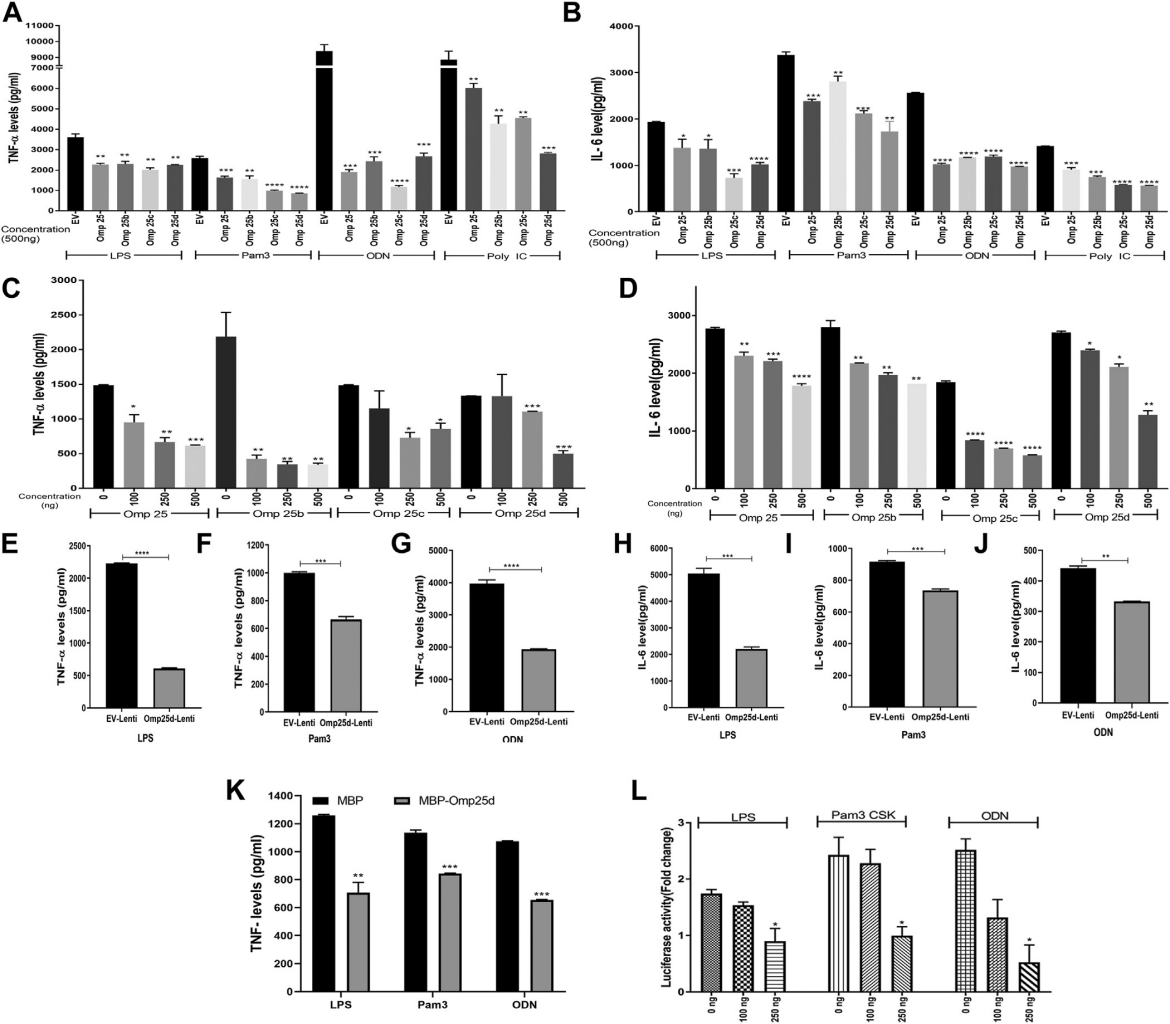
**Outer membrane protein 25 (Omp25) and its variants suppress TLR-mediated activation of NF-κB and production of proinflammatory cytokines.***A* and *B*, Omp25 and its variants suppress proinflammatory cytokines induced by various TLR ligands. RAW264.7 cells were transfected with plasmids expressing MYC-Omp25 or its variants (500 ng) for 24 h, followed by treatment with LPS (300 ng/ml) or Pam3-CSK4 (300 ng/ml) or ODN (1 μg/ml) or poly:IC (20 μg/ml), followed by quantification by ELISA to determine the levels of TNF-α (*A*) and IL-6 (*B*). *C* and *D*, Omp25 and its variants suppress LPS-induced proinflammatory cytokines in a dose-dependent manner. RAW264.7 cells were transfected with various concentrations (100, 250, and 500 ng) of plasmid expressing MYC-Omp25 or its variants for 24 h, followed by inducing the transfected cells with LPS (300 ng/ml) for 5 h. The levels of TNF-α and IL-6 were quantified by ELISA. *E*–*J*, RAW264.7 cells were transduced with lentiviral particles harboring Omp25d or empty vector for 48 h, followed by induction with LPS (300 ng/ml), Pam3-CSK4 (300 ng/ml), or ODN (1 μg/ml). Subsequently, the supernatants were collected, and levels of TNF-α and IL-6 were quantified using ELISA. *K*, recombinant MBP-Omp25d protein suppresses the production of TNF-α by macrophages. RAW264.7 cells were treated with 5 μg of MBP-Omp25 or MBP alone for 3 h, followed by induction with LPS, Pam3-CSK4, or ODN. Subsequently, the supernatants were collected, and the levels of TNF-α and IL6 were quantified using ELISA. *L*, Omp25 attenuates TLR-induced activation of NF-κB. RAW264.7 cells were cotransfected with MYC-Omp25 (0, 100, and 250 ng), pNF-κB–Luc (300 ng), and pRL-TK (50 ng) plasmids. Twenty-four hours post-transfection, the cells were stimulated with the appropriate TLR ligands such as LPS (200 ng/ml) for TLR4, Pam3-CSK4 (200 ng/ml) for TLR2, and ODN (1 μg/ml) for TLR9 for 16 h. The activation of NF-κB was determined by quantifying the luciferase activity in the cell lysates. The fireﬂy luciferase activity was normalized with renilla luciferase, and the data were expressed as mean fold induction over unstimulated controls. The data were analyzed using GraphPad Prism software, and statistical significance was determined using unpaired *t* test. The data are presented as mean SD from at least three independent experiments (∗*p* ≤ 0.05; ∗∗*p* ≤ 0.01; ∗∗∗*p* ≤ 0.001; and ∗∗∗∗*p* ≤ 0.0001). IL-6, interleukin 6; LPS, lipopolysaccharide; MBP, maltose-binding protein; ODN, oligodenucleotide; Poly:IC, polyinosinic–polycytidylic acid; TLR, Toll-like receptor; TNF-α, tumor necrosis factor alpha.

*Brucella* spp. are known to suppress the production of proinflammatory cytokines in the host for their chronic persistence. The effector proteins, *viz*. Omp25 and TcpB, have been reported to play an essential role in the attenuation of proinflammatory cytokines in the host. Since the TLR signaling pathways play a major role in the induction of proinflammatory cytokines, we wished to examine whether Omp25 and its variants interfere with the TLR signaling pathways to attenuate production of proinflammatory cytokines in macrophages. RAW264.7 cells were transfected with plasmid expressing Omp25 or its variants, and the overexpression was confirmed by quantitative RT–PCR (qRT–PCR) and immunoblotting (Fig. S1, *A* and *B*). Subsequently, the mouse macrophages overexpressing Omp25 and its variants were induced with TLR ligands, lipopolysaccharide (LPS) (TLR4), Pam3-CSK4 (TLR2), ODN (oligodenucleotide) (TLR9), and polyinosinic–polycytidylic acid (poly:IC; TLR3), followed by evaluation of TNF-α, IL-6, and IFN-β levels by ELISA and qPCR. We found that Omp25 and its variants could efficiently suppress the LPS, Pam3-CSK4, ODN, and poly:IC-induced expression and secretion of TNF-α, IL-6, and IFN-β in macrophages (Figs. 1, *A* and *B* and S2, *A*–*C*). Furthermore, we transfected RAW264.7 cells with increasing concentrations of Omp25 or its variants, followed by induction with LPS and quantification of TNF-α and IL-6 by ELISA and qPCR. Omp25 or its variants could efficiently suppress the production of proinflammatory cytokines in a dose-dependent manner (Fig. 1, *C* and *D* and S2, *D* and *E*). The experimental data suggest that the Omp25 and its variants attenuate production of proinflammatory cytokines that are mediated by TLR2, TLR3, TLR4, and TLR9. In addition, we transducted RAW264.7 cells with lentiviral particles harboring the Omp25d expression plasmid or empty vector, followed by confirming the expression of Omp25d by qPCR and immunoblotting using maltose-binding protein (MBP)-Omp25d-immunized mice serum (Fig. S1, *C* and *D*). Subsequently, the transducted cells were treated with LPS, Pam3-CSK4, or ODN, and the levels of TNF-α and IL-6 were analyzed using ELISA and qPCR. We observed suppression of TNF-α and IL-6 in the Omp25d-expressing cells compared with the cells transduced with lentivirus harboring the empty vector (Figs. 1, *E*–*J* and S2, *F* and *G*). Furthermore, we examined the levels of TNF-α in the macrophages treated with the recombinant Omp25 protein. RAW264.7 cells were treated with MBP-Omp25d or MBP alone, followed by inducing the cells with LPS, Pam3-CSK4, or ODN and quantification of TNF-α by ELISA and qPCR. In agreement with the previous observation, the cells treated with MBP-Omp25d showed diminished TNF-α levels compared with the cells treated with MBP alone (Figs. 1*K* and S2*H*). The NF-κB regulates the expression of various proinflammatory cytokines in macrophages. Therefore, we examined whether Omp25 interferes with the activation of NF-κB mediated by TLRs. We performed a luciferase-based reporter assay to analyze the effect of one of the Omp25 variants, Omp25d, on TLR2/4/9-mediated NF-κB activation. RAW264.7 cells were cotransfected with various concentrations of Omp25 and reporter plasmids. Subsequently, the cells were stimulated with the appropriate TLR ligands, such as LPS for TLR4, Pam3-CSK4 for TLR2, and ODN for TLR9, overnight. The activation of NF-κB was determined by quantifying the luciferase activity in the presence or the absence of Omp25. We observed a dose-dependent suppression of luciferase activity by Omp25 (Fig. 1*L*). Since the NF-κB mediates expression of many proinflammatory cytokines, its inhibition can affect the production of proinflammatory cytokines (45).

### Omp25 interacts with the TLRs and their adapter proteins

**Figure 2:**
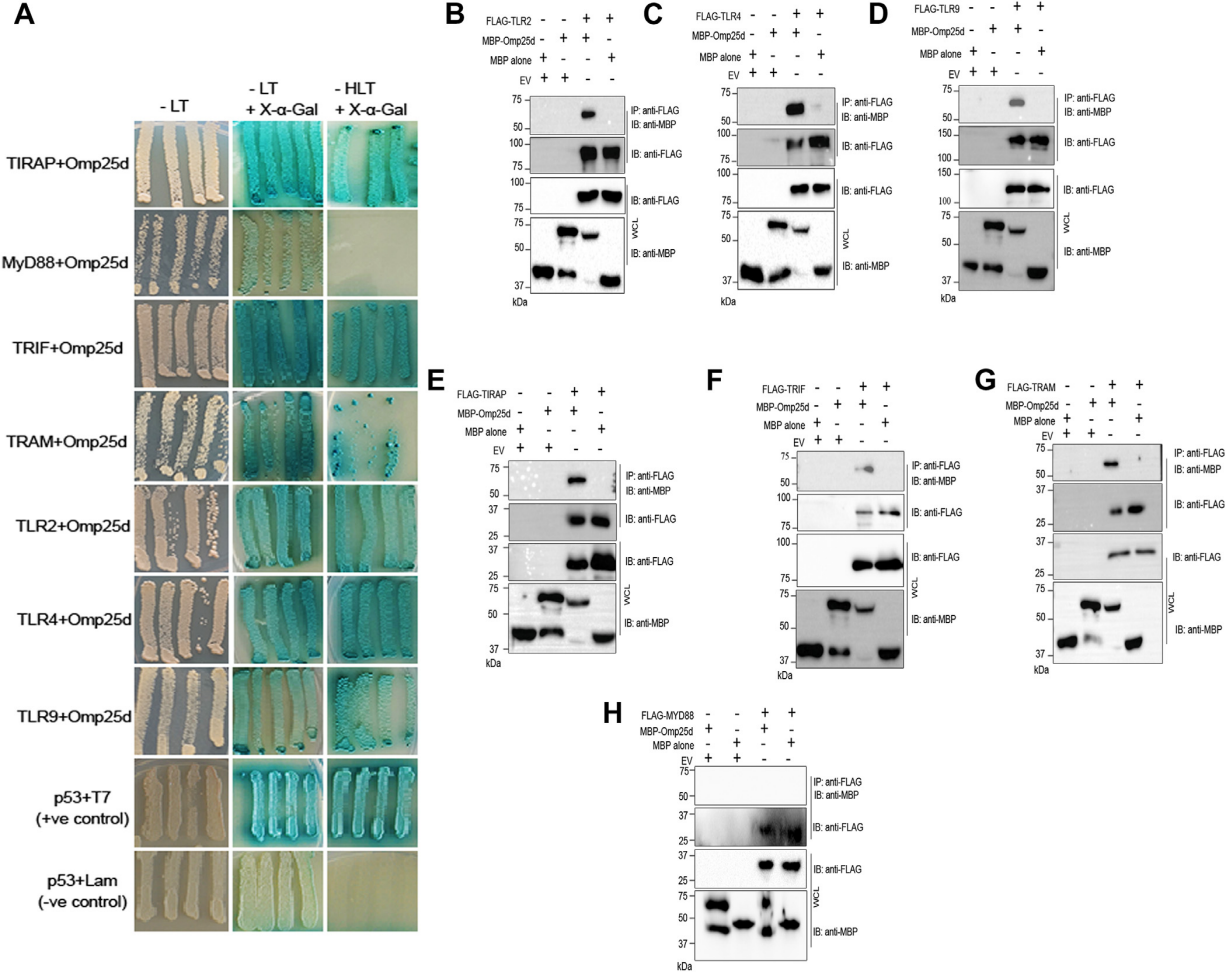
**Outer membrane protein 25 (Omp25) interacts with TLRs and their adaptor proteins except for MYD88.***A*, yeast two-hybrid assays showing the interaction of Omp25 with TLRs and adaptor proteins. AH109 yeast strain was transformed with bait plasmid harboring Omp25d fused with DNA-binding domain (BD) and the prey plasmid harboring TIRAP/TLR2/TLR4/TLR9/TRIF/MYD88 fused with the DNA activation domain (AD). Both the bait and prey plasmids were selected on SD/-Leu/-Trp amino acid dropout medium, followed by analyzing their interaction by streaking the yeast on the same media with X-α-Gal or on the triple amino acid dropout media, SD/-His/-Leu/-Trp containing X-α-Gal. The growth of yeast with *blue color* on the amino acid dropout media indicates the positive interaction. AH109 yeast harboring the plasmid expressing lamin fused with BD and T-antigen fused with AD served as the negative control, whereas the yeast carrying p53 fused with BD and T-antigen fused with AD served as the positive control. *B*–*H*, confirmation of interaction between Omp25 and TLRs or adaptor proteins by coimmunoprecipitation (co-IP). The lysate of HEK293T cells overexpressing FLAG-tagged TLR2/TLR4/TLR9/TIRAP/TRIF/TRAM/MYD88 was mixed with purified MBP-Omp25d or MBP alone, followed by IP of FLAG-tagged proteins using the anti-FLAG antibody and immunoblotting. The co-IP of MBP-Omp25d with FLAG-tagged TLRs or adaptor proteins suggests the positive interaction. No interaction was observed between MBP-Omp25 and FLAG-MYD88. The immunoblots are representative of two independent experiments. AD, activation domain; BD, binding domain; HEK293T, human embryonic kidney 293T cell line; MBP, maltose-binding protein; TLR, Toll-like receptor; WCL, whole-cell lysate.

We found that Omp25 and its variants attenuate the secretion of proinflammatory cytokines mediated by various TLRs. It is possible that Omp25 interacts with the TLRs or other key proteins in the TLR signaling pathway to interfere with the signaling cascade. The activation of TLRs leads to the recruitment of TIR domain–containing adaptor proteins to the cytoplasmic side of TLRs, and this molecular assembly initiates the signaling cascade. Therefore, we sought to examine whether Omp25 interacts with the TLRs or their adaptor proteins using yeast two-hybrid assays and coimmunoprecipitation (co-IP). For yeast two-hybrid assays, the AH109 yeast strain was transformed with bait plasmid harboring Omp25d fused with DNA-binding domain and the prey plasmid containing TLR2/TLR4/TLR9/TIRAP/MYD88/TRIF/TRAM fused with the DNA activation domain. The bait and prey plasmids in the yeast transformants were selected using SD/-Leu/-Trp amino acid dropout medium. The interaction between the bait and prey plasmids was examined by plating the yeast on SD/-His/-Leu/-Trp triple KO media containing X-α-Gal. The growth of blue-colored yeast colonies on a triple amino acid dropout medium indicates a positive interaction between the bait and prey plasmid. We observed the growth and blue color development in the yeast that harbor Omp25d and TLR2/TLR4/TLR9/TIRAP/TRIF/TRAM suggesting a positive interaction between the Omp25d and the indicated prey proteins (Fig. 2*A*). However, we did not observe the growth of yeast harboring Omp25d and MYD88 on triple amino acid dropout media, which indicates lack of interaction between these proteins (Fig. 2*A*).

Next, we performed co-IP to confirm the experimental data obtained from the yeast two-hybrid assays. Human embryonic kidney 293T (HEK293T) cells were transfected with the expression plasmid harboring FLAG-tagged TLR2/TLR4/TLR9/TIRAP/TRIF/TRAM/MYD88. Subsequently, the HEK293T lysates containing the overexpressed TLRs or adaptor proteins were mixed with MBP-tagged Omp25d or MBP alone, followed by IP of FLAG-tagged proteins using anti-FLAG antibody. The immunoprecipitated proteins were then analyzed by SDS-PAGE and immunoblotting. The membrane was probed with anti-MBP antibody to detect MBP-Omp25d in the immunoprecipitated samples. We observed co-IP of Omp25d with TLR2/TLR4/TLR9/TIRAP/TRIF/TRAM indicating the positive interaction (Fig. 2, *B*–*G*). In agreement with the yeast two-hybrid assay, we did not observe co-IP of Omp25d with MYD88, indicating the absence of interaction between Omp25d and MYD88 (Fig. 2*H*). MBP alone was not coimmunoprecipitated with TLRs or adaptor proteins, which indicates the specificity of the interactions (Fig. 2, *B*–*G*). Collectively, our data show that Omp25d interacts with TLR2, TLR4, TLR9 and the adaptor proteins TIRAP and TRIF but not with MYD88.

### Omp25 promotes degradation of TLRs and adaptor proteins

**Figure 3:**
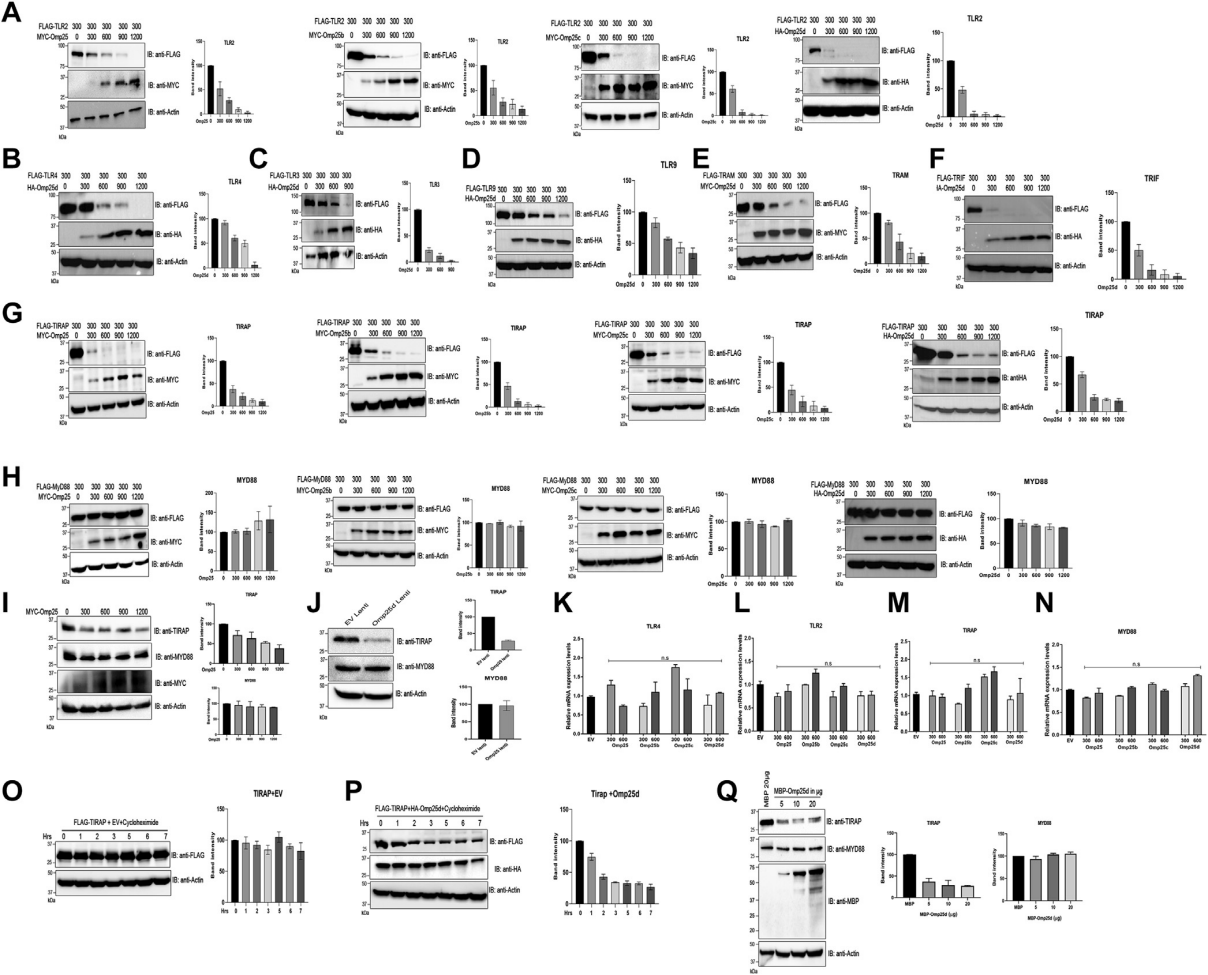
**Outer membrane protein 25 (Omp25) induces degradation of TLRs and their adaptor proteins except for MYD88.***A*–*H*, HEK293T cells were cotransfected with indicated concentrations of FLAG-tagged TLR2/TLR3/TLR4/TLR9/TIRAP/MYD88/TRIF/TRAM and MYC/HA-tagged Omp25 or its variants. Cells were lysed 24 h post-transfection, followed by immunoblotting. Omp25 and its variants induced degradation of TLRs and their adaptor proteins except for MYD88. *I*, Omp25 promotes the degradation of endogenous TIRAP in macrophages. RAW264.7 cells were transfected with indicated concentrations of MYC-Omp25 expressing plasmid. Twenty-four hours post-transfections, cells were harvested, followed by immunoblotting and detection of endogenous TIRAP and MYD88. *J*, the levels of endogenous TIRAP and MYD88 in macrophages transduced with lentivirus particles harboring Omp25 expression construct. RAW264.7 cells were transducted with lentiviral particles harboring Omp25d or empty vector. Fourty-eight hours post-transduction, cells were collected, followed by immunoblotting and detection of endogenous TIRAP and MYD88. Omp25d delivered through the lentivirus promoted the degradation of TIRAP. *K*–*N*, Omp25 does not affect the expression of its target proteins. RAW264.7 cells were transfected with indicated concentrations of Omp25 or its variants. Twenty-four hours post-transfections, total RNA was extracted from the cells, followed by cDNA synthesis and qPCR analysis. The transcript levels of endogenous TLR2, TLR4, TIRAP, or MYD88 were not altered in the presence of Omp25 or its variants. *O* and *P*, pulse-chase analysis of TIRAP degradation by Omp25d. HEK293T cells were cotransfected with FLAG-TIRAP and HA-Omp25d or empty vector for 24 h, followed by treatment with cycloheximide. Subsequently, the cells were harvested at the indicated time points, lysed, and subjected to immunoblotting. The gradual degradation of FLAG-TIRAP was detected in the presence of HA-Omp25d with increasing time points in cycloheximide-treated cells. *Q*, the recombinant MBP-Omp25d protein induces degradation of endogenous TIRAP. RAW264.7 cells were treated with purified MBP-Omp25d or MBP alone for 5 h. Subsequently, the cells were lysed and subjected to immunoblotting to detect MBP-Omp25d, endogenous TIRAP or MYD88, and actin. The recombinant MBP-Omp25d protein induced degradation of endogenous TIRAP but did not affect the level of MYD88. Actin served as the loading control for all the immunoblots. *Right panel* of each blot indicates the densitometry of TLRs or their adaptor protein bands, which were normalized with actin. The immunoblots are representative of three independent experiments. cDNA, complementary DNA; EV, empty vector; HA, hemagglutinin; HEK293T, human embryonic kidney 293T cell line; MBP, maltose-binding protein; qPCR, quantitative PCR; TLR, Toll-like receptor.

Targeted degradation of key proteins in the TLR signaling pathways has been reported to be an intrinsic cellular mechanism to negatively regulate TLR signaling to dampen the excess inflammatory responses (46, 47, 48). The TcpB protein of *Brucella* has been reported to exploit this host regulatory mechanism where it promotes ubiquitination and degradation of the TLR2/4 adaptor protein, TIRAP (36). Given that Omp25 interacts with the TLRs and their adaptor proteins, we wished to examine whether Omp25 induces degradation of its interacting partners. HEK293T cells were cotransfected with increasing concentrations of hemagglutinin (HA)/MYC-tagged Omp25 or its variants and equal concentrations of FLAG-tagged TLR2/TLR3/TLR4/TLR9/TIRAP/MYD88/TRIF/TRAM, followed by cell lysis and immunoblotting. The immunoblots were probed with anti-FLAG antibody to detect FLAG-tagged TLR or adaptor proteins. We observed the degradation of TLR2, TLR3, TLR4, TLR9, TRAM, TIRAP, and TRIF with increasing concentrations of Omp25 or its variants (Figs. 3, *A*–*G* and S3, *A*–*D*). Omp25 or its variants did not induce the degradation of MYD88 (Fig. 3*H*), which was in agreement with our previous observation where no interaction was detected between Omp25 and MYD88. Furthermore, we wished to examine the endogenous levels of TIRAP and MYD88 in the presence of Omp25. To analyze this, RAW264.7 cells were transfected with increasing concentrations of MYC-Omp25. Twenty-four hours post-transfection, the cells were lysed, followed by immunoblotting to detect the endogenous levels of TIRAP and MYD88. We observed an enhanced degradation of endogenous TIRAP in the presence of Omp25, whereas MYD88 levels remained unaltered (Fig. 3*I*). To confirm the data further, we analyzed the endogenous levels of TIRAP and MYD88 in RAW264.7 cells transduced with lentivirus particles harboring the Omp25d expression plasmid. We observed reduced levels of TIRAP in the Omp25d-expressing cells, whereas the levels of MYD88 remained unaffected by Omp25d (Fig. 3*J*).

It is possible that Omp25 may affect the expression of TLRs and adaptor proteins at the transcriptional level that can also result in a decreased level of TLRs and adaptor proteins in the presence of Omp25. To examine this, we analyzed the mRNA levels of TIRAP and MYD88 in HEK293T cells overexpressing equal concentrations of FLAG-TIRAP or FLAG-MYD88 and increasing concentrations of Omp25 variants. The mRNA levels of TIRAP and MYD88 remained constant with the increasing concentrations of Omp25 (Fig. S3, *E* and *F*). To verify the data further, we transfected RAW264.7 cells with increasing concentrations of Omp25 or its variants, followed by quantifying the endogenous levels of TLR2, TLR4, TIRAP, and MYD88. We observed no significant changes in the expression levels of TLR2, TLR4, TIRAP, and MYD88 with increasing concentrations of Omp25, indicating that Omp25 does not affect the gene expression of its targets (Fig. 3, *K*–*N*). To further confirm that Omp25 affects at the post-translational level, we performed a pulse-chase analysis to examine the degradation of TIRAP by Omp25d. HEK293T cells were cotransfected with HA-Omp25d or empty vector and FLAG-TIRAP, followed by treating the cells with cycloheximide that inhibits the protein synthesis. The cells were harvested at various time points, followed by immunoblotting and probing with anti-FLAG antibody to detect the level of FLAG-TIRAP. We observed an enhanced degradation of TIRAP in the presence of Omp25d with increasing time points (Fig. 3, *O* and *P*). To examine whether the recombinant Omp25 protein retains the ability to degrade adaptor proteins, we treated RAW264.7 cells with purified MBP or MBP-Omp25d for 5 h and examined the endogenous levels of TIRAP and MYD88. We observed an enhanced degradation of TIRAP in the presence of MBP-Omp25d, whereas MYD88 level remains unchanged as observed before. Treatment with MBP alone did not induce any degradation of TIRAP or MYD88 indicating the specific effect of Omp25d (Fig. 3*Q*). Collectively, our data imply that Omp25 and its variants promote degradation of TLR2, TLR3, TLR4, TLR9, TIRAP, and TRIF to attenuate the TLR signaling and the production of proinflammatory cytokines.

### Omp25 induces ubiquitination of TLRs and adaptor proteins

**Figure 4:**
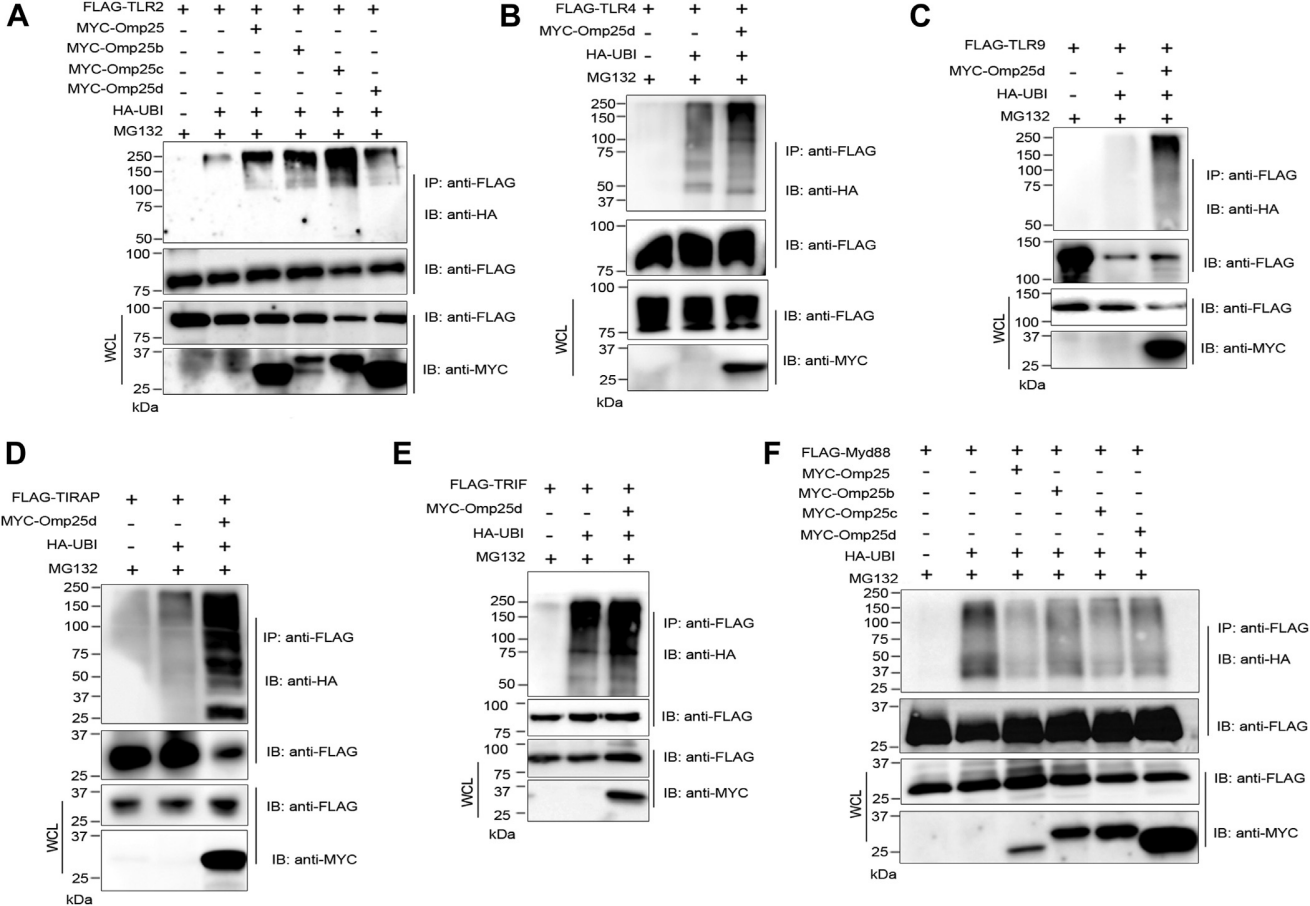
**Outer membrane protein 25 (Omp25) promotes ubiquitination of TLRs and adaptor proteins.***A*–*F*, HEK293T cells were cotransfected with various combinations of MYC-Omp25 or its variants and FLAG-tagged TLR2/TLR4/TLR9/TIRAP/TRIF/MYD88 and HA-ubiquitin as indicated in the panels. The transfected cells were treated with the proteasome inhibitor, MG132. Twenty-four hours post-transfections, the cells were lysed, followed by immunoprecipitation of FLAG-tagged TLRs or adaptor proteins. The membranes were probed with anti-HA antibody to detect HA-ubiquitin–conjugated TLRs or adaptor proteins. An enhanced ubiquitination was detected with TLR2, TLR4, TLR9, TIRAP, and TRIF but not with MYD88 in the presence of Omp25 or its variants. Immunoblots are representative of two independent experiments. HA, hemagglutinin; HEK293T, human embryonic kidney 293T cell line; TLR, Toll-like receptor.

Ubiquitination is a prerequisite for the proteasome-mediated degradation of eukaryotic proteins (49). Given that Omp25 promoted enhanced degradation of TLRs and their adaptor proteins, we sought to examine whether Omp25 induces their ubiquitination. HEK293T cells were cotransfected with expression plasmids harboring Omp25 or its variants and FLAG-tagged TLRs or adaptor proteins and HA-ubiquitin. The cells were treated with the proteasome inhibitor, MG132, followed by lysis and immunoblotting. The ubiquitin moieties that are conjugated to the TLRs and their adaptor proteins were detected using anti-HA antibody. We found an enhanced ubiquitination of TLR2, TLR4, TLR9, TIRAP, and TRIF in the presence of Omp25 or its variants (Fig. 4, *A*–*E*). In agreement with the previous findings, Omp25 did not affect the ubiquitination status of MYD88 (Fig. 4*F*). The experimental data indicate that Omp25 and its variants promote enhanced ubiquitination of TLRs and their adaptor proteins that can lead to the proteasome-mediated degradation of these proteins.

### Omp25 affects the endogenous level of TIRAP in *Brucella*-infected macrophages

**Figure 5:**
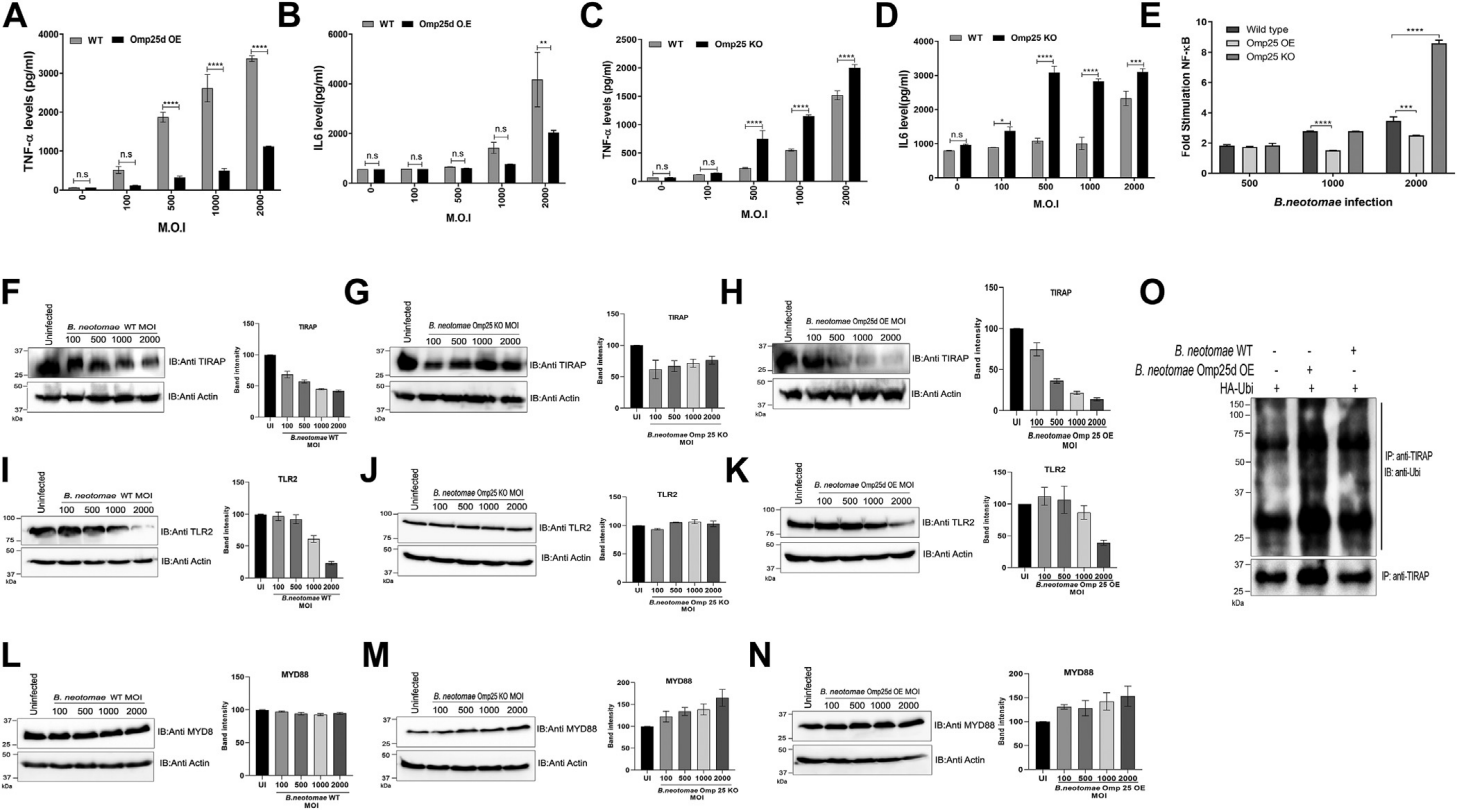
**Overexpression or deletion of outer membrane protein 25 (Omp25) in *Brucella* affects the production of proinflammatory cytokines in macrophages.***A*–*D*, RAW264.7 cells were infected with various MOIs of WT or Omp25 KO or Omp25 overexpressing *Bacillus neotomae*. Levels of TNF-α (*A* and *C*) and IL-6 (*B* and *D*) in *B. neotomae*-infected RAW264.7 cells were examined at 24 h postinfection. *E*, overexpression of Omp25 attenuates NF-κB activation in macrophages. RAW264.7 cells were transfected with pNF-κB–Luc and pRL-TK reporter plasmids. Twenty-four hours post-transfections, the cells were infected with increasing MOI of WT, Omp25 KO, and *B. neotomae*-overexpressing Omp25d. Twenty-four hours postinfection, the cells were lysed and luciferase activity was measured in the cell lysates. The fireﬂy luciferase activity was normalized with renilla luciferase, and the data were expressed as mean fold induction over uninfected controls. *F*–*N*, overexpression or deletion of Omp25 in *Brucella* affects the endogenous levels of TIRAP and TLR2. Mouse macrophages were infected with various MOIs of WT/Omp25 KO or Omp25 overexpressing *B. neotomae*. Twenty-four hours postinfection, cells were lysed and subjected to immunoblotting. The blots were probed with anti-TIRAP, anti-TLR2, and anti-MYD88 antibodies to detect the endogenous levels of TIRAP, TLR2, and MYD88, respectively. Actin served as the loading control. *Right panel* indicates the densitometry of the TIRAP, TLR2, and MYD88, which was normalized with actin. Immunoblots are representative of two independent experiments. *O*, Omp25 promotes ubiquitination of TIRAP in the *Brucella*-infected macrophages. RAW264.7 cells were transfected with HA-ubiquitin for 12 h, followed by infection with WT or *B. neotomae* overexpressing HIS-Omp25d. Twenty-four hours postinfection, the cells were lysed, followed by immunoprecipitation of TIRAP using anti-TIRAP antibody and immunoblotting. The membrane was probed with anti-ubiquitin antibody and antimouse IgG-HRP to detect ubiquitin-conjugated TIRAP. The data were analyzed using GraphPad Prism software, and statistical significance was determined using an unpaired *t* test. The data are presented as mean SD from at least two independent experiments (∗*p* ≤ 0.05; ∗∗*p* ≤ 0.01; and ∗∗∗*p* ≤ 0.001). Immunoblots are representative of two independent experiments. HA, hemagglutinin; HRP, horseradish peroxidase; IgG, immunoglobulin G; IL-6, interleukin 6; MOI, multiplicity of action; TLR, Toll-like receptor; TNF-α, tumor necrosis factor alpha; WCL, whole-cell lysate.

Given that Omp25 and its variants induce ubiquitination and degradation of TLRs and adaptor proteins upon transient expression of Omp25, we sought to examine the effect of Omp25 in the *Brucella*-infected macrophages. To analyze this, we generated Omp25 KO and Omp25d-overexpressing *Bacillus neotomae* strains. We confirmed the deletion of Omp25 and overexpression of Omp25d in *B. neotomae* by qPCR (Fig. S4, *A* and *B*). Next, RAW264.7 cells were infected with various multiplicity of infections (MOIs) of WT/Omp25 KO or Omp25d-overexpressing *B. neotomae* strains, followed by analyzing the levels of proinflammatory cytokines. In agreement with our previous findings, overexpression of Omp25d in *B. neotomae* enhanced the suppression of TNF-α and IL-6 in the infected macrophages (Fig. 5, *A* and *B*). In contrast, we observed a diminished suppression of TNF-α and IL-6 by Omp25 KO *B. neotomae* compared with WT (Fig. 5, *C* and *D*). These studies show that Omp25 plays an important role in the downregulation of proinflammatory cytokines in the *Brucella*-infected macrophages. Next, we analyzed the levels of NF-κB activation in the macrophages infected with WT/Omp25 KO or Omp25d-overexpressing *B. neotomae* strains. RAW264.7 cells were transfected with NF-κB reporter plasmids, followed by infection with increasing MOI of *B. neotomae* strains and luciferase reporter assay. We observed an enhanced suppression of NF-κB activation in the macrophages infected with *B. neotomae*-overexpressing Omp25d strain compared with WT. In contrast, a diminished suppression of NF-κB activation was observed in macrophages infected with Omp25 KO *B. neotomae* (Fig. 5*E*). The experimental data indicate that Omp25 attenuates NF-κB activation in the *Brucella*-infected macrophages resulting in the suppression of proinflammatory cytokines.

Next, we examined the interaction between Omp25 and TIRAP–TLR2 in the macrophages infected with *B. neotomae*. RAW264.7 cells were infected with *B. neotomae*-overexpressing HIS-tagged Omp25d, followed by the pull-down of HIS-Omp25d using nickel–nitrilotriacetic acid resin and immunoblotting. Subsequently, the blot was probed with anti-TIRAP or anti-TLR2 antibodies to detect the respective proteins. We observed the presence of TIRAP and TLR2 in the pull-down samples indicating the interaction of these proteins with Omp25d (Fig. S5*A*). Furthermore, we mixed the lysates of RAW264.7 and *B. neotomae*-overexpressing HIS-Omp25d, followed by IP of TIRAP and detection of HIS-Omp25d. We observed co-IP of Omp25d along with TIRAP confirming the interaction between these proteins (Fig. S5*B*). Next, we analyzed the endogenous level of TIRAP, MYD88, and TLR2 in the macrophages infected with WT or genetically modified *B. neotomae*. Macrophages were infected with various MOIs of WT/Omp25-KO or Omp25d-overexpressing *B. neotomae*, followed by lysing the cells and analyzing the endogenous level of TIRAP, MYD88, and TLR2 by immunoblotting. We observed a gradual decrease in the levels of TIRAP or TLR2 in the macrophages infected with increasing MOIs of WT *B. neotomae*. The Omp25 KO *B. neotomae* failed to promote degradation of TIRAP or TLR2, whereas Omp25d-overexpressing *B. neotomae* caused an enhanced degradation of TIRAP or TLR2 as compared with the WT in the infected macrophages (Fig. 5, *F*–*K*). The levels of MYD88 remained unaltered in the samples, indicating no effect of Omp25 on MYD88 degradation (Fig. 5, *L*–*N*). Next, we examined the ubiquitination of TIRAP in the macrophages infected with WT or *B. neotomae*-overexpressing HIS-Omp25d. RAW264.7 cells transfected with HA-ubiquitin, followed by infection with *B. neotomae* and IP of TIRAP. We observed enhanced ubiquitination of TIRAP in the macrophages infected with *B. neotomae*-overexpressing HIS-Omp25d (Fig. 5*O*). Collectively, our experimental data suggest that Omp25 promotes ubiquitination and degradation of its target proteins in the *Brucella*-infected macrophages to attenuate the production of proinflammatory cytokines.

### The purified recombinant MBP-Omp25d protein was internalized by macrophages through endocytosis

**Figure 6:**
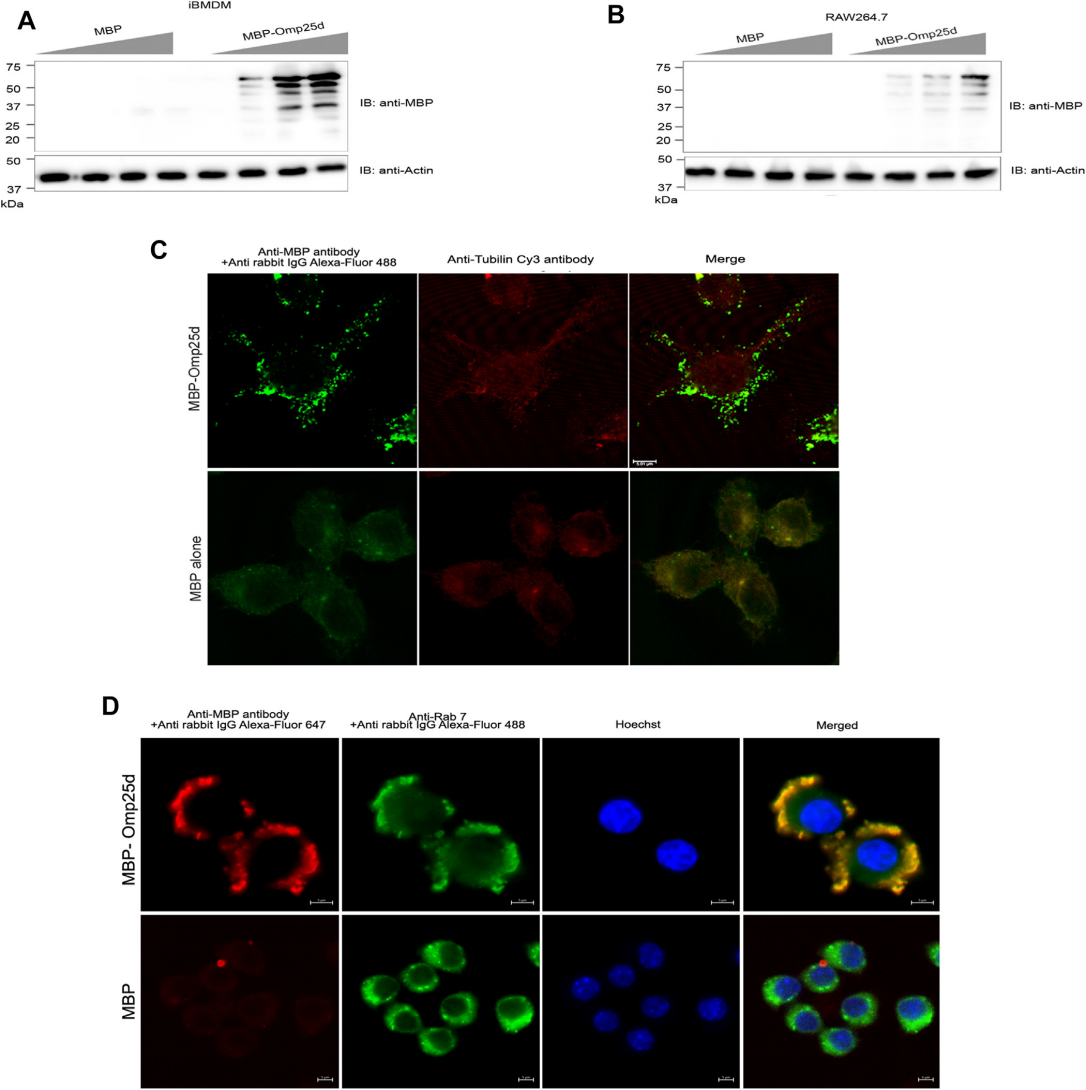
**Outer membrane protein 25 (Omp25) is internalized by macrophages through endocytosis.***A* and *B*, cellular uptake of purified recombinant MBP-Omp25d protein by macrophages. Immortalized BMDMs (*A*) or RAW264.7 (*B*) cells were treated with increasing concentrations (0, 25, 50, and 100 μg) of MBP-Omp25d or MBP protein alone for 5 h. Subsequently, the cells were washed and treated with 0.1% trypsin to remove the extracellular membrane-bound proteins, followed by lysis and immunoblotting. The blot was probed with anti-MBP antibody to detect the internalized MBP-Omp25d. Actin served as the loading control. The immunoprobing indicated multiple bands probably because of the cleavage of MBP-Omp25d inside the macrophages. The immunoblots are representatives of two independent experiments. *C*, detection of internalized MBP-Omp25d by confocal microscopy. RAW264.7 cells were treated with 100 μg/ml of purified MBP-Omp25d or MBP alone for 5 h. Subsequently, cells were washed and treated with 0.1% trypsin to remove the extracellular proteins. The cells were then fixed, permeabilized, and stained with anti-MBP antibody, followed by anti-rabbit Alexa Flour-488 as secondary antibody to detect the internalized MBP-Omp25d. Cy3-labeled antitubulin antibody was used to stain the tubulin. Scale bar represents 5 μm. *D*, subcellular localization of MBP-Omp25 and Rab7. RAW264.7 cells were treated with 100 μg/ml of purified MBP-Omp25d or MBP alone for 5 h. Subsequently, cells were washed and treated with 0.1% trypsin to remove the extracellular membrane-bound proteins. The cells were then fixed, permeabilized, and stained with anti-MBP and anti-Rab7 antibodies, followed by anti-rabbit Alexa Flour-488 secondary antibody (Rab7) and Alexa Flour-647 secondary antibody (MBP-Omp25d). Rab7 was detected in punctate bodies along with MBP-Omp25d. Scale bar represents 5 μm. Images are representative of three independent experiments. BMDM, bone marrow–derived macrophage; MBP, maltose-binding protein.

Omp25 was reported to be released into the culture supernatant of *Brucella*, and the concentrated culture supernatant could efficiently suppress LPS-induced secretion of TNF-α in macrophages (39). This suggests that Omp25 may be internalized by mammalian cells to inhibit the proinflammatory signaling pathways. Therefore, we sought to examine whether Omp25d is internalized by the mammalian cells. RAW264.7 macrophages were treated with increasing concentrations of purified MBP-Omp25d or MBP alone. Subsequently, the cells were washed thoroughly and treated with trypsin–EDTA to remove the extracellular MBP-Omp25d or MBP. The cells were then lysed and subjected to immunoblotting. The blot was probed with anti-MBP antibody to detect the MBP-Omp25d or MBP. We observed accumulation of MBP-Omp25d in the macrophages in a dose-dependent manner (Fig. 6, *A* and *B*). The absence of intracellular MBP in the samples treated with MBP alone indicates that the cell permeability is conferred by Omp25d (Fig. 6, *A* and *B*). To further confirm these data, we stained the MBP-Omp25d or MBP alone–treated macrophages with anti-MBP antibody, followed by Alexa 488-conjugated anti-rabbit immunoglobulin G (IgG). Cy3-labeled anti-tubulin antibody was used to stain tubulin. We observed the presence of MBP-Omp25d inside the macrophages in distinct puncta (Fig. 6*C*). To examine whether macrophages internalize MBP-Omp25 through endocytosis, we treated RAW264.7 cells with MBP-Omp25d or MBP, followed by staining the cells with anti-Rab7 antibody to detect Rab7, which is an endosomal surface marker. The cells were stained with anti-MBP antibody to detect the internalized MBP-Omp25d protein. Alexa 488-conjugated antimouse IgG and Alexa 647-conjugated anti-rabbit IgG secondary antibodies were used for Rab7 and MBP-Omp25, respectively. We observed colocalization of MBP-Omp25 with Rab7-labeled punctuated bodies, suggesting its internalization through endocytosis (Fig. 6*D*). Collectively, our experimental data suggest that the Omp25d is endocytosed into the macrophages to access its intracellular targets for attenuating the TLR signaling.

## Discussion

The Omps contribute to the invasion, intracellular multiplication, and modulation of host immune responses in *Brucella*. Therefore, Omps are considered as potential candidates for development of vaccines and diagnostic assays for brucellosis. Approximately, 75 different types of Omps are reported on the surface of *Brucella* (50). The major Omps of *Brucella* are classified into three groups, according to their molecular weights. Group I includes Omp10 and Omp19; group II constitutes Omp2a and Omp2b porin proteins, and group III is composed of Omp25 and Omp31. There are seven homologous Omps in group III that are Omp25 and its three variants: Omp25b, Omp25c, and Omp25d; Omp31 and Omp31b, and Omp22 (38, 43, 49). The average amino acid sequence identity among the Omp25 and its variants ranges from 45% to 68% with many conserved motifs. Omp25b is absent in *Brucella ovis* and *B. abortus* because of deletion of a 15 kb region from the chromosomal DNA (51). Omp25, Omp25c, and Omp25d are highly conserved among the classical species of *Brucella* (Fig. S6) (51). The Omp25 has been reported to attenuate production of proinflammatory cytokines, but the immunomodulatory properties of Omp25b, Omp25c, and Omp25d remain obscure. Our studies revealed that Omp25 and its variants could efficiently suppress production of proinflammatory cytokines mediated by various TLRs. Furthermore, we found that Omp25 and its variants promote proteasome-mediated degradation of key proteins in the TLR signaling pathways to attenuate the NF-κB activation and to suppress the expression of proinflammatory cytokines.

Ubiquitination and subsequent degradation of key signaling proteins constitute an efficient mechanism to negatively regulate TLR signaling. This intrinsic cellular mechanism prevents the aberrant activation of TLRs to maintain cellular homeostasis. The negative regulators are often induced by TLR agonists that suppress further activation of TLRs through the negative feedback mechanism. Many suppressor of cytokine signaling (SOCS) proteins are induced by TLR ligands, including LPS, and these proteins negatively regulate TLR signaling pathways by targeting various signaling mediators (47, 52, 53). SOCS-1 is an E3 ubiquitin ligase that promotes enhanced ubiquitination and degradation of TLR2/4 adaptor protein TIRAP, thereby blocking the signaling cascade from these receptors (47). SOCS-1, SOCS-2, SOCS-3, and TRIM38 have been reported to target TRAF-6 for proteasome-dependent degradation to negatively regulate the production of proinflammatory cytokines (54, 55, 56). The E3 ubiquitin ligase, Triad3A, induces ubiquitination and degradation of TLR4 and TLR9 to regulate the signaling cascade (46). The peptidyl-prolyl isomerase 1 has been reported to induce polyubiquitination and degradation of IRF-3 to attenuate expression of type I interferons (57).

The negative regulation of TLRs is essential for controlling the excess production of proinflammatory cytokines in macrophages and dendritic cells. However, a controlled release of proinflammatory cytokines is required for activation and maturation of various antigen-presenting cells for establishing adaptive immune responses against the invaded pathogens. Many bacterial pathogens secrete effector proteins that suppress the induction of proinflammatory cytokines, thereby preventing the activation of cell-mediated immune responses. The NleC of enteropathogenic and enterohemorrhagic *E. coli* and CT441 of *Chlamydia* promote proteolytic cleavage of p65 subunit of NF-κB to dampen the host inflammatory responses (58, 59). The TIR domain–containing effector proteins of bacterial pathogens have been reported to interfere with the TLR or IL-1R signaling pathways to attenuate the proinflammatory responses. TlpA of *Salmonella enteric* serovar enteritidis suppresses NF-κB activation, which is mediated by TLR4, MYD88, and IL-1R (18). TcpC of *E. coli* inhibits TLR2- and TLR4-mediated activation of NF-κB and proinflammatory cytokines by interacting with the adaptor protein, MYD88 (21). The TIR domain–containing protein from *Y. pestis*, YpTdP, has also been reported to suppress IL-1R- and TLR4-mediated signaling to dampen the host immune responses (60).

*Brucella* species encode three effector proteins with immunomodulatory properties, *viz*. TcpB, BtpB, and Omp25. Both TcpB and BtpB are TIR domain–containing proteins with proven anti-inflammatory properties. TcpB has been reported to attenuate TLR2/4 and inflammasome-mediated expression of proinflammatory cytokines (21, 35, 36). TcpB induces proteasome-mediated degradation of the TLR2/4 adaptor protein, TIRAP, and the inflammatory caspases for suppressing the production of proinflammatory cytokines in macrophages (35, 36). The BtpB effector protein is also involved in modulation of host innate immune responses during the infection of *Brucella* (61). Omp25 has been reported to play an important role in the invasion, proliferation, and chronic persistence of *Brucella* in the host. *Brucella* species secrete or release Omp25 into the invaded cells to facilitate their intracellular proliferation and immunomodulation (49, 62). Intriguingly, our experimental data indicated that the recombinant Omp25d is efficiently internalized by the macrophages through endocytosis.

The two-component regulatory system of *Brucella*, BvrR/BvrS, has been reported to regulate the expression of Omp25 and its variants at the transcriptional level (41, 63). Therefore, the defective intracellular replication of BvrR/BvrS mutant may be attributed to the deficiency of Omp25 expression. It has been reported that the Omp25 KO *B. melitensis*, *B. abortus*, and *B. ovis* displayed an attenuated phenotype in the infected mice (64). A defective splenic colonization was observed with these mutants because of their inefficiency to invade and modulate the host immune responses. A differential expression of Omp25 variants has been reported in many species of *Brucella*. Even though all the Omp25 family members possess the ability to suppress production of proinflammatory cytokines, their requirement for virulence appears to differ. It has been demonstrated that the deletion of Omp25c in *B. ovis* did not affect the bacterial virulence, whereas the deletion of Omp25d affected invasion, intracellular replication, and mice infection of *B. ovis* (38). Furthermore, a compensatory mechanism has also been reported to exist in the Omp25 gene family as the deletion of one gene in the Omp25 family resulted in an enhanced expression of other Omp25 members (38). In agreement with this, Omp25 and its variants exhibited similar properties irrespective of their limited sequence identity.

Omp25 plays an essential role in subverting the host innate and adaptive immune responses to facilitate chronic persistence of *Brucella*. Even though the immunomodulatory properties of Omp25 have been established by various studies, its mechanism of action remains obscure. Studies have shown that Omp25 suppressed TLR-induced IL-12 production through upregulation of programmed death-1 protein, which in turn induced the expression of miR-155, miR-23b, and miR-21-5P in the Omp25-expressing or *B. suis*-infected cells (40). MicroRNA 21-5P and miR-23b directly affected the expression of IL-12, whereas miR-155 suppressed IL-12 through the inhibition of NF-κB activation that results in downregulation of TAB2 (TGF-beta-activated kinase 1 and MAP3K7-binding protein 2) (40). Omp25 has been reported to bind to SLAMF1 (signaling lymphocytic activation molecule family member 1) receptor on dendritic cells that affected nuclear translocation of NF-κB and expression of proinflammatory cytokines, which in turn prevented the activation of dendritic cells (65). Our experimental data revealed that Omp25 induces degradation of TLRs and adaptor proteins to attenuate TLR-mediated secretion of proinflammatory cytokines. The cytokines, IL12 and TNF-α, serve as two central proinflammatory cytokines in mammals (66). Both IL-12 and TNF-α play a major role in the activation and maturation of antigen-presenting cells such as macrophages and dendritic cells. Therefore, attenuation of IL-12 and TNF-α can lead to a defective antigen presentation to T lymphocytes. In agreement with this, Omp25-mediated suppression of TNF-α prevented the maturation of *Brucella*-infected dendritic cells, and an exogenous addition of TNF-α restored their maturation and antigen presentation (67). Furthermore, IL-12 plays an important role in the differentiation of naïve T-helper cells to IFN-γ producing Th1-type cells, which is essential for the clearance of *Brucella* (68, 69). Therefore, suppression of these proinflammatory cytokines can lead to a defective adaptive immune response, which can facilitate chronic persistence of *Brucella* in the host. Furthermore, inter-regulation of TNF-α and IL-12 is required for balancing the macrophage function in innate and adaptive immune responses (70). Our studies also revealed that Omp25 attenuated expression of IFN-β, which has been reported to promote and regulate various immune responses, macrophage reprogramming, and enhanced bacterial clearance (71, 72).

In summary, we found that Omp25 and its variants suppress production of TLR-mediated proinflammatory cytokines in macrophages. Furthermore, we showed that Omp25 interacted with various TLRs and adaptor proteins and induced their ubiquitination and degradation to attenuate the expression of proinflammatory cytokines. TLRs detect various PAMPs to induce the production of proinflammatory cytokines that result in the development of adaptive immune responses against the invaded pathogen. Our studies revealed that the Omp25 targets multiple TLRs and their adaptor proteins to effectively suppress the induction of proinflammatory cytokines. This may contribute to the defective adaptive immune responses observed in the *Brucella*-infected hosts that is accounted for the chronic persistence of *Brucella*. The information derived from our studies may help to design novel preventive strategies for brucellosis. Our studies also revealed the key mediators and the cellular processes that negatively regulate TLR signaling cascade to prevent their aberrant activation. This information may help to develop novel therapeutics for various infectious and inflammatory disorders.

## Experimental procedures

### Cell culture and transfections

RAW264.7, HEK293T (American Type Culture Collection) cells, and immortalized bone marrow–derived macrophages (iBMDMs; a gift from Petr Broz, University of Lausanne) were cultured in Dulbecco’s modified Eagle's medium (DMEM; Sigma) supplemented with 10% fetal bovine serum (Sigma) and 1× penicillin–streptomycin solution (Gibco). The iBMDMs were differentiated using 25 ng/ml of mouse colony-stimulating factor (R&D Systems) in DMEM. All the cells were grown in the incubator at 37 °C with 10% humidity and 5% CO_2_. Transfection of RAW264.7 and iBMDMs with plasmid DNA was performed using Xfect transfection reagent (Takara Bio) according to the manufacturer’s instructions. TurboFect transfection reagent (Thermo Scientific) was used to transfect HEK293T cells according to the manufacturer’s instructions.

### Overexpression of Omp25 and its variants in macrophages and quantification of TLR-induced cytokines

RAW264.7 (1 × 10^5^/ml) cells were seeded in 24-well plates and allowed to adhere overnight. Subsequently, the cells were transfected with various concentrations (100, 250, and 500 ng per well) of eukaryotic expression plasmid (pCMV-MYC) harboring Omp25 or its variants. Twenty-four hours post-transfection, the cells were treated with various inducers of TLRs, such as LPS (TLR4; 300 ng/ml) or Pam3-CSK4 (TLR2; 300 ng/ml) for 2 h or ODN (TLR9; 1 μg/ml) overnight or poly:IC (TLR3; 20 μg/ml) for 24 h. Subsequently, the cells and culture supernatants were harvested for analyzing the production of proinflammatory cytokines using qPCR or ELISA. For qPCR, total RNA was isolated from the cells using RNAiso Plus extraction reagent (Takara Bio), followed by complementary DNA synthesis using PrimeScript first strand complementary DNA synthesis kit (Takara Bio) and qPCR analysis (Bio-Rad). The levels of proinflammatory cytokines in the culture supernatants were quantified using Quantikine Duo ELISA kit (R&D Systems) as per the manufacturer’s protocol.

To generate lentivirus particles harboring Omp25d expression plasmid, Omp25d was cloned into the lentivirus expression plasmid, pMSCV-PIG (a gift from David Bartel; Addgene plasmid #21654). To prepare lentivirus particles harboring pMSCV-PIG-Omp25, HEK293FT cells (1 × 10^6^ in 6-well plates) were cotransfected with packaging vector psPAX2 (a gift from Didier Trono, Addgene plasmid #12260) and the envelope vector, pMD2.G (a gift from Didier Trono, Addgene plasmid #12259), and pMSCV-PIG-Omp25d or empty vector. Seventy-two hours post-transfection, the culture supernatant containing lentivirus particles was collected and clarified by centrifugation and passed through a 0.45 μm syringe filter (Millipore). To overexpress Omp25d using lentivirus, RAW264.7 (1 × 10^6^) cells were transducted with lentivirus particles harboring the Omp25d expression plasmid. Forty-eight hours after transduction, the lentivirus-transduced cells were analyzed by qPCR using Omp25d-specific primers to confirm the overexpression. To examine the expression of Omp25d by immunoblotting, we generated MBP-Omp25d immune sera in mice. Mice (n = 3) were injected with 40 μg of MBP-Omp25d protein with Freund’s complete adjuvant (Sigma; 1:1 ratio) intraperitoneally after the prebleeds. About 14 days postimmunization, blood was collected from the immunized mice through the retro-orbital route, followed by the preparation of serum. RAW264.7 cells transduced with lentivirus harboring the empty vector or Omp25d-expressing plasmid were lyzed and subjected to immunoblotting. The membrane was probed with pooled prebleed or MBP-Omp25d-immunized serum, followed by horseradish peroxidase (HRP)–conjugated anti-mouse IgG.

To determine the proinflammatory cytokine levels in the lentivirus-transducted cells, RAW264.7 macrophages were treated with lentiviral particles harboring Omp25d or empty vector for 48 h. Subsequently, the cells were washed with 1× PBS and induced with various TLR ligands as described before. The supernatants and cells were collected and processed for ELISA and qPCR to determine the levels of proinflammatory cytokines. The experiments were performed independently at least three times, and the result was analyzed using GraphPad Prism software (GraphPad Software, Inc).

### NF-κB reporter assay

In order to analyze TLR4-induced NF-κB activation in the presence of Omp25, RAW264.7 cells were seeded into 12-well plates at a density of 0.5 × 10^6^ cells per well. The cells were then cotransfected with different doses of MYC-Omp25 (100 and 250 ng), pNF-κB–Luc (300 ng), and pRL-TK (50 ng) plasmids. The total amount of DNA was made constant by adding the empty vector (pCMV-MYC). Twenty-four hours post-transfection, the cells were stimulated with LPS (TLR4 ligand; 200 ng/ml) or Pam3-CSK4 (TLR2 ligand; 200 ng/ml) or ODN (TLR9 ligand; 1 μg/ml) overnight. The cells were then lysed, and the luciferase activity was assayed using the Dual-luciferase reporter assay system (Promega) according to the manufacturer’s protocols. The fireﬂy luciferase activity was normalized with renilla luciferase, and the data were expressed as mean fold induction over unstimulated controls. To examine the NF-κB activation in *Brucella*-infected macrophages, RAW264.7 cells were transfected with pNF-κB-Luc (1 μg) and pRL-TK (200 ng) plasmids. Twenty-four hours post-transfections, the cells were infected with WT/Omp25 KO/*B. neotomae*-overexpressing Omp25d. About 24 h postinfection, the luciferase activity was analyzed as described before. The assays were performed in triplicate, and the experiments were repeated thrice.

### Yeast two-hybrid screening

Omp25d gene was amplified from *B. neotomae* and cloned into the yeast two-hybrid vector, pGBKT7 (Clontech) in fusion with the GAL4 DNA-binding domain to generate pBD-Omp25d (bait). FLAG-tagged TIRAP (gifted by Dr Douglas Golenbock), TLR2, TLR4 (a gift from Ruslan Medzhitov; Addgene plasmid #13087), TLR9, TRIF (a gift from Kate Fitzgerald & Tom Maniatis; Addgene plasmid #41550), TRAM (a gift from Kate Fitzgerald & Doug Golenbock; Addgene plasmid #41551), and MYD88 (gifted by Dr Douglas Golenbock) plasmids were used to amplify respective TLR or adaptor protein genes and cloned into pGADT7 vector in fusion with DNA activation domain to generate prey plasmids. Next, pBD-Omp25d and the individual prey plasmid were introduced into the yeast strain, AH109 (Clontech) using lithium acetate/single-stranded carrier DNA/polyethylene glycol method. The diploid yeast colonies were selected on SD agar with double amino acid dropout (-Leu/-Trp) medium. The interaction with Omp25d and TIRAP/TLR2/TLR4/TLR9/TRIF/TRAM/MYD88 was assayed on SD agar with triple amino acid dropout medium (-His/-Leu/-Trp) (Clontech) containing X-α-Gal (Clontech).

### Co-IP

HEK293T cells (1 × 10^6^) were transfected with pCMV-FLAG expression plasmid harboring TIRAP/TLR2/TLR4/TLR9/TRIF/TRAM using TurboFect reagent in 6-well plates. Forty-eight hours post-transfection, cells were lysed at 4 °C in the lysis buffer containing 20 mM Tris (pH 8.0), 150 mM NaCl, 1% Triton X-100, 1 mM EDTA, and 1× Protease Inhibitor Cocktail (Pierce) (IP buffer), followed by clarification of the lysate by centrifugation at 12,000 rpm for 20 min. Subsequently, the lysates harboring individual FLAG-tagged TLRs or adaptor proteins were mixed with purified MBP-Omp25d or MBP alone. Next, the lysates were precleared with Protein G plus agarose beads (Santa Cruz Biotechnology) and mixed with 5 μg of anti-FLAG antibody (Sigma), followed by overnight incubation at 4 °C on a rotator. Next, Protein G plus agarose beads were added to the samples and rotated further for 2 h at 4 °C. Subsequently, agarose beads were washed three times with 1× IP buffer (20 mM Tris [pH 8.0], 150 mM NaCl, and 1% Triton X-100) and resuspended in 30 μl of 2× SDS sample buffer (Bio-Rad). Samples were then boiled for 10 min, followed by SDS-PAGE and immunoblotting. The membrane was probed with HRP-conjugated anti-MBP antibody (NEB; 1:5000 dilution) to detect MBP-tagged protein and HRP-conjugated anti-FLAG antibody (1:5000 dilution) to detect FLAG-tagged proteins. To determine the interaction between Omp25 and endogenous TIRAP in the macrophages, the lysates of RAW264.7 cells and *B. neotomae*-overexpressing Omp25 were mixed and incubated overnight with anti-TIRAP antibody (5 μg; CST) at 4 °C on a rotator. Subsequently, the TIRAP was immunoprecipitated and subjected to immunoblotting. The membrane was probed with anti-HIS-HRP antibody (R&D Systems; 1:5000 dilution) to detect coimmunoprecipitated HIS-Omp25d. Anti-TIRAP primary antibody, followed by HRP-conjugated anti-rabbit IgG (CST; 1:3000 dilution), was used to detect TIRAP. The uninfected RAW264.7 cell lysate was used as the negative control. To pull-down Omp25 from the *Brucella*-infected cells, RAW264.7 cells were infected with *B. neotomae*-overexpressing Omp25d for 36 h, followed by harvesting the cells. The cells were lysed using IP buffer and incubated overnight with nickel–nitrilotriacetic acid resin (100 μl) at 4 °C on a rotator. Subsequently, the resin was washed thrice with 1× PBS, boiled with 2× Laemmli dye, and processed for immunoblotting. The membranes were probed with anti-TIRAP and anti-TLR2 primary antibodies, followed by HRP-conjugated anti-rabbit IgG antibody to detect the respective proteins. The HIS-Omp25d was detected using anti-HIS-HRP antibody. Super Signal West Pico Chemiluminescent Substrate (Pierce) was used as per the manufacturer’s protocol. The luminescent signal was acquired using a Chemi-documentation system (Syngene).

### Cotransfections to examine protein degradation

HEK293T cells (0.5 × 10^6^) cotransfected with equal concentration of pCMV-FLAG expression plasmids harboring TIRAP/MYD88/TRIF/TRAM/TLR2/TLR3/TLR4/TLR9 and increasing concentrations of pCMV-HA or MYC-tagged Omp25 or its variants in 12-well plates. Twenty-four hours post-transfection, cells were lysed using radioimmunoprecipitation assay (RIPA) buffer, and protein concentration was estimated using Bradford reagent (Sigma). Equal amount of protein samples was loaded on 12% SDS-PAGE gel, followed by immunoblotting. The membrane was probed with HRP-conjugated anti-FLAG antibody (1:5000 dilution; Sigma) to detect FLAG-tagged TLRs or adaptor proteins, HRP-conjugated anti-MYC antibody (1:5000 dilution; Sigma) for detecting MYC-Omp25 or its variants, and HRP-conjugated anti-HA antibody (1:5000 dilution; Sigma) for detecting HA-Omp25 or its variants. To examine the endogenous level of TIRAP and MYD88 in the presence of Omp25, RAW264.7 cells (0.5 × 10^6^) were transfected with increasing concentrations (300, 600, 900, and 1200 ng per well) of pCMV-MYC-Omp25. Total DNA was made constant by adding pCMV-MYC empty vector. Twenty-four hours post-transfection, the cells were harvested, followed by lysis and immunoblotting. The endogenous TIRAP or MYD88 was detected using primary antibody against TIRAP or MYD88 (CST; 1:1000 dilution), followed by HRP-conjugated antimouse IgG (CST; 1:3000 dilution). Actin served as the loading control. To examine the endogenous levels of TIRAP and MYD88 in the lentivirus-transduced cells, RAW264.7 cells were transducted with lentiviral particles harboring MSCV-PIG-Omp25d and pMSCV-PIG empty vector. Forty-eight hours post-transduction, the cells were harvested and processed for immunoblotting. The endogenous levels of TIRAP and MYD88 were detected as described before. Actin served as the loading control.

### Pulse-chase analysis of protein degradation

HEK293T cells (0.5 × 10^6^) were transfected with pCMV-FLAG harboring TIRAP alone or in combination with pCMV-MYC-Omp25d. Twenty-hours post-transfection, the cells were treated with cycloheximide, followed by collection of cells at 0, 1, 2, 3, 5, 6, and 7 h post-treatment. The cells were then lysed and subjected to immunoblotting.

### *In vivo* ubiquitination assay

HEK293T cells (1 × 10^6^) were cotransfected with 0.5 μg of pCMV-MYC-Omp25d or their variants, 1.5 μg of pCMV-FLAG harboring TIRAP/TLR2/TLR4/TLR9/TRIF/MYD88, and 1 μg of pCMV-HA-Ubiquitin (gifted by Dr Shigeki Miyamoto) with various combinations. DNA concentration was maintained at 3 μg using the empty vector. Eight hours post-transfections, the cells were treated with proteasome inhibitor MG132 (Sigma) at a concentration of 10 μM overnight. After 24 h of transfections, the cells were treated again with 20 μM MG132 for 2 h. Subsequently, the cells were washed with PBS and lysed in 300 μl of denaturing lysis buffer containing 20 mM Tris–HCI (pH 7.4) and 1% SDS. Cell lysates were transferred into individual Eppendorf tubes and boiled for 10 min, followed by centrifugation at 13,000 rpm for 15 min for the clarification of the lysate. Next, the cell lysates were diluted with a buffer containing 20 mM Tris–HCl (pH 7.5), 150 mM NaCl, 2% Triton X-100, and 0.5% NP-40. Subsequently, 5 μg of anti-FLAG antibody was added into the lysates and kept overnight at 4 ^o^C at rotation. The FLAG-tagged TLRs or adaptor proteins were immunoprecipitated as described before. The samples were resolved on SDS-PAGE, followed by immunoblotting. The membrane was probed with HRP-conjugated anti-HA and anti-FLAG antibodies (1:5000 dilution; Sigma) to detect HA-ubiquitin and FLAG-tagged proteins, respectively. Omp25d in whole-cell lysate was detected using an HRP-conjugated anti-MYC antibody (1:5000 dilution; Sigma). To determine the ubiquitination of endogenous TIRAP in the *Brucella*-infected macrophages, RAW264.7 cells were transfected with HA-ubiquitin for 12 h, followed by infection with WT or *B. neotomae*-overexpressing HIS-Omp25 at an MOI of 1:3000. Twenty-four hours postinfection, the cells were lysed in the denaturing lysis buffer and performed IP of TIRAP using the anti-TIRAP antibody (5 μg; CST) and immunoblotting as described before. The membrane was probed with the antiubiquitin antibody (1:1000 dilution; CST) to detect ubiquitinated TIRAP and the anti-TIRAP antibody to detect the immunoprecipitated TIRAP. The uninfected RAW264.7 cells were used as the negative control.

### Generation of Omp25 gene KO

To construct a suicide plasmid for deletion of Omp25 gene by homologous recombination, a three-way ligation technique was used. One kilobyte fragments, upstream and downstream of Omp25 gene, were amplified from the chromosomal DNA of *B. neotomae*. The forward and reverse primers for the amplification of upstream fragments harbored KpnI and BamHI restriction enzyme sites, respectively. The BamHI and XbaI sites were added in the forward and reverse primers, respectively, for amplifying the downstream fragment. The kanamycin expression cassette from the pUC-kan-LoxP plasmid was released with BamHI restriction digestion. The pZErO-1 plasmid (Invitrogen) digested with KpnI and XbaI was used as the suicide vector. Next, the upstream fragment, kanamycin cassette, and the downstream fragment were ligated into KpnI and XbaI of pZErO-1 by three-way ligation. The plasmid was transformed into *E. coli* (DH5α), and the positive clones were confirmed by selecting on zeocin and kanamycin plates. Subsequently, the Omp25KO plasmid was introduced into *B. neotomae* (ATCC23459) by electroporation. The upstream and downstream fragments in the KO plasmid recombine with the similar sequences on the chromosomal DNA of *B. neotomae*, leading to the replacement of Omp25 gene with the kanamycin expression cassette. The transformed *B. neotomae* colonies growing on kanamycin and zeocin, which indicate a single recombination event and the resulting insertion of the KO plasmid into the chromosomal DNA, were discarded. The zeocin-sensitive and kanamycin-resistant Omp25 KO *B. neotomae* colonies were selected and confirmed further by PCR.

### Generation of *B. neotomae*-overexpressing Omp25d

In order to overexpress Omp25d in *B. neotomae*, the Omp25d gene was cloned into the *Brucella* expression plasmid, pNSTrcD at the BamHI and PstI sites, in-frame with the 6× histidine tag. *B. neotomae* harboring pNSTrcD-Omp25 was selected on chloramphenicol (40 μg/ml) plates. The overexpression of Omp25d in *B. neotomae* was confirmed further by qRT–PCR.

### Macrophage infection with *B. neotomae* and mutants

RAW264.7 or iBMDM cells were seeded in 12-well plate format overnight in antibiotic-free DMEM supplemented with 10% fetal bovine serum, followed by infection with *B. neotomae* or Omp25 KO *B. neotomae* or *B. neotomae*-overexpressing Omp25d at an MOI of 100, 500, 1000, and 2000 for 90 min. Subsequently, the cells were washed three times with PBS and treated with gentamicin (30 μg/ml) for 30 min to kill the extracellular bacteria. The cells were then washed twice with PBS and maintained in complete DMEM containing gentamicin (3 μg/ml), followed by collection of cell supernatants at 24 h postinfection for quantification of proinflammatory cytokines by ELISA. For determining endogenous protein degradation, cells were collected and lysed using RIPA buffer, and protein concentration was estimated using Bradford reagent (Sigma). Equal amount of protein was loaded on SDS-PAGE gel, followed by immunoblotting. Membranes were probed with anti-TIRAP, anti-MYD88, or anti-TLR2 antibodies (1:1000 dilution; Sigma), followed by HRP-conjugated anti-rabbit IgG (1:3000 dilution; CST).

### Overexpression and purification of Omp25d

The Omp25d gene was amplified from the chromosomal DNA of *B. neotomae*, followed by cloning the gene into pMALC4G (NEB) prokaryotic expression vector in-frame with MBP. The resulting expression plasmid, pMAL-Omp25d, was confirmed by restriction digestion and sequencing. For overexpression and purification of Omp25d, 1 L of LB medium containing ampicillin (100 mg/ml) was inoculated with 0.1% of overnight grown *E. coli* (BL21) cells harboring pMAL-Omp25d or pMAL-C4G empty plasmid. The culture was grown at 37 °C with shaking, followed by induction of protein expression with 1 mM IPTG when the absorbance reached 0.6 at 600 nm. After the induction, cells were grown at 25 °C for 5 h with shaking. Subsequently, the bacterial cells were collected by centrifugation at 10,000 rpm for 10 min and washed with PBS. Next, the bacterial pellet was resuspended in the sonication buffer containing 50 mM Tris–HCl (pH 8.0), 1 M NaCl, 1 mM EDTA, and 1× Protease Inhibitor Cocktail (Thermo Fisher) and sonicated for 20 min (45 s ON and 15 s OFF). The sonicated supernatant was then clarified by centrifugation at 16,000*g* for 20 min. Next, the clarified supernatant was passed through the column harboring amylose resin (NEB), followed by washing the column with sonication buffer and then with the sonication buffer containing decreasing concentrations of NaCl (750, 500, 250, and 100 mM). Protein elution was performed with an elution buffer containing 50 mM Tris–HCl (pH 8.0) and 30 mM maltose. The eluted protein was concentrated using a Centricon protein concentrator (Millipore) and dialyzed in a buffer containing 50 mM Tris–HCl, 100 mM NaCl, and 10% glycerol. The residual LPS was removed from the purified proteins using endotoxin removal spin columns according to the manufacturer’s protocol (Thermo Scientific). The concentration of MBP-Omp25d or MBP was estimated using Bradford reagent (Sigma). The purified proteins were aliquoted and stored in a −80 °C freezer after snap freezing in liquid nitrogen for further experiments.

### Protein internalization

Immortalized BMDMs or RAW264.7 cells were treated with increasing concentrations (0, 25, 50, and 100 μg) of MBP-Omp25d or MBP protein alone for 5 h. Subsequently, the cells were washed with PBS three times, followed by treatment with 0.1% trypsin–EDTA for 5 min to digest the extracellular MBP-Omp25d or MBP, if any. The cells were then washed with PBS three more times, followed by cell lysis with RIPA buffer and immunoblotting. HRP-conjugated anti-MBP antibody (NEB) was used to detect MBP-Omp25d or MBP protein. Actin served as the loading control.

### Confocal microscopy

RAW264.7 cells (1 × 10^6^) were seeded into 30 mm glass bottom dishes and allowed to adhere overnight. Subsequently, the cells were treated with 20 μg/ml of MBP-Omp25d or MBP alone for 5 h in fresh culture media. The cells were then washed twice with PBS and treated with 0.1% trypsin–EDTA for 5 min. Subsequently, the cells were washed and treated with 4% paraformaldehyde solution in PBS and incubated for 20 min to fix the cells. After fixation, the cells were washed three times with PBS and permeabilized with 0.1% Triton X-100 for 5 min. The cells were then washed three times with PBS and incubated with a blocking solution containing 1% bovine serum albumin and 50 mM NH_4_Cl in PBS for 30 min. Subsequently, the cells were washed and stained with anti-MBP antibody (1:200 dilution; NEB), followed by anti-rabbit IgG-Alexa Flour 488 (1:200 dilution; CST). Cy3-labeled anti-tubulin antibody was used for staining tubulin. To examine the internalization of MBP-Omp25 through endocytosis, MBP-Omp25 was stained with anti-MBP antibody (1:200 dilution), and Rab 7 was stained with anti-Rab7 antibody (1:200 dilution). Antimouse Alexa Flour 647 (1:200 dilution; CST) and anti-rabbit IgG-Alexa Flour 488 (1:200 dilution) were used as secondary antibodies for MBP-Omp25 and Rab7, respectively. Subsequently, the cells were washed and mounted in ProLong Gold Antifading reagent with 4′,6-diamidino-2-phenylindole (Invitrogen) and analyzed using a confocal microscope (Leica).

### Ethics statement

Female BALB/c mice of 6 to 8 weeks old (20–25 g; three mice) were housed at the small animal experimentation facility of the National Institute of Animal Biotechnology with food and water ad libitum. The experimental protocols were approved by the Institutional Animal Ethics Committee (approval number: IAEC/NIAB/2023/34/GKR).

## Data availability

All the data supporting this study are available in the article and supporting information.

## Supporting information

This article contains supporting information.

## Conflict of interest

The authors declare that they have no conflicts of interest with the contents of this article.
