# Supplementary material for: Outer membrane protein 25 of Brucella suppresses TLR-mediated expression of proinflammatory cytokines through degradation of TLRs and adaptor proteins
Source: J Biol Chem. 2023 Sep 29;299(11):105309. doi: 10.1016/j.jbc.2023.105309 (PMC10641269; doi:10.1016/j.jbc.2023.105309)
Supplement: Supporting Figure S1 [file mmc1.docx]

**Supporting information Figure 1**

**
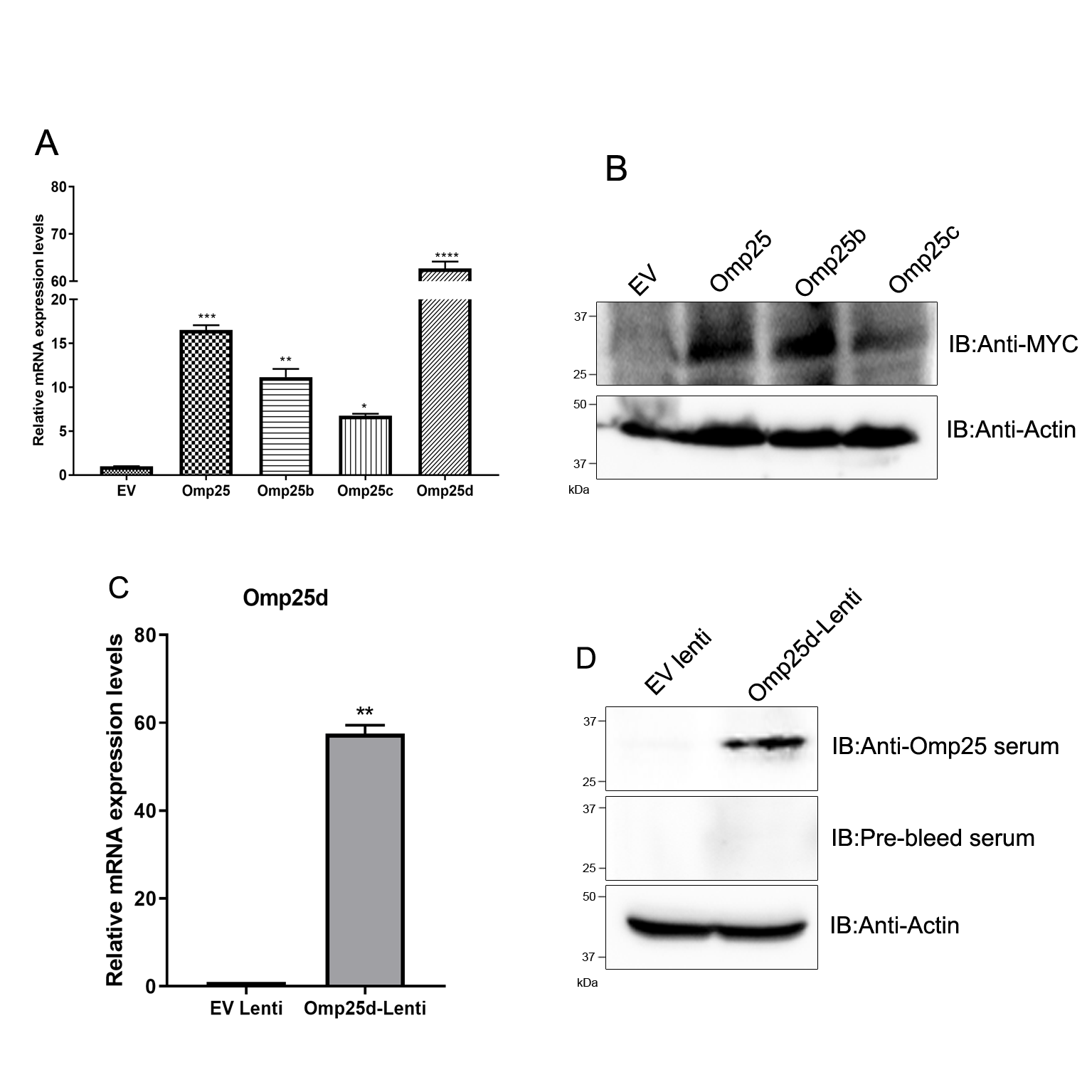
**

**Supporting information 1 (A-B): Transfection efficiency of Omp25 and variants plasmid in RAW264.7 cells**. RAW264.7 cells were transfected with plasmids expressing Myc-Omp25, Myc-Omp25b, Myc-Omp25c and HA-Omp25 (1 ug) for 24 hours. Cells were harvested to isolate RNA, followed by cDNA preparation and quantitative PCR for mRNA expression levels of Omp25 or its variants. The data were normalized with GAPDH, and relative mRNA expression was quantified in comparison with EV.For immunoblotting, cells were collected 36 hours post-transfection, followed by lysis and immunoblotting using anti-Myc antibody. Actin was used as a loading control. **(C-D) The expression of Omp25d in RAW264.7 cells transduced with the lentiviral particles by qRT-PCR (C) and Immoblotting (D) .** **(C)**RAW264.7 cells were transducted with lentiviral particles harboring Omp25dexpression plasmid or empty vector. Forty-eight hours post-transduction, the cells were collected in TRIzol and processed for RNA isolation, cDNA synthesis, and qPCR analysis to determine the mRNA expression levels of Omp25d. GAPDH was used to normalize the data and the relative mRNA expression was determined with respect to the cells transduced with lentivirus harboring the empty vector. **(D)** Confirmation of expression of Omp25 through immunobloting, EV and Omp25 expressing-lenti-transducted cell lysates were processed for immunoblotting and membranes probed with MBP-Omp25d immunized mice serum followed by anti-mouse IgG-HRP. Pre-bleed serum served as negative control.
