# Supplementary material for: Outer membrane protein 25 of Brucella suppresses TLR-mediated expression of proinflammatory cytokines through degradation of TLRs and adaptor proteins
Source: J Biol Chem. 2023 Sep 29;299(11):105309. doi: 10.1016/j.jbc.2023.105309 (PMC10641269; doi:10.1016/j.jbc.2023.105309)
Supplement: Supporting Figure S2 [file mmc2.docx]

**
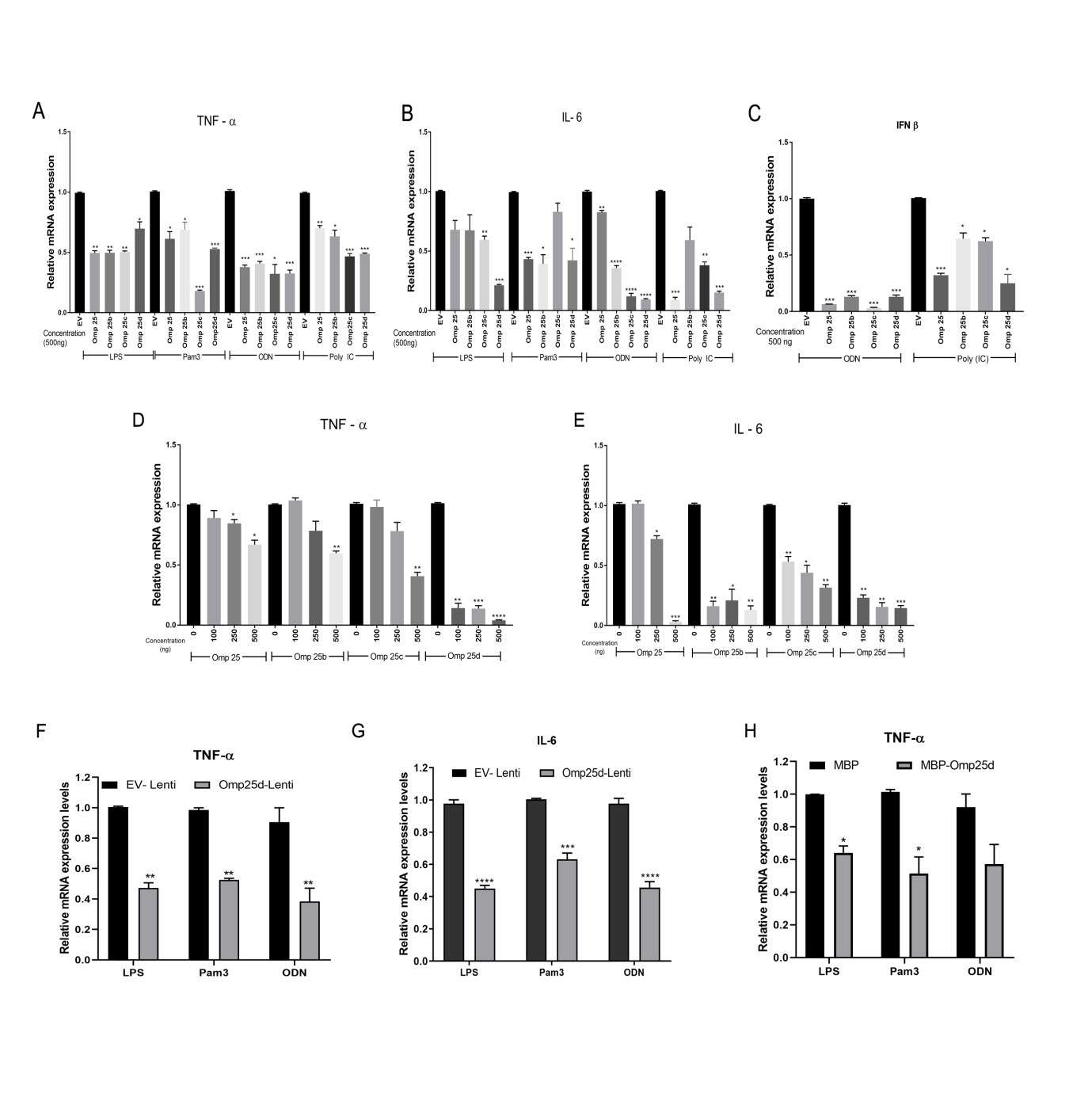
Supporting information Figure 2**

**Supporting information 2 (A-C): Omp25 and its variants suppress the production of pro-inflammatory cytokines induced by various TLR ligands.** RAW264.7 cells were transfected with plasmids expressing MYC-Omp25 or its variants (500 ng) for 24 hours, followed by treatment with LPS (TLR4; 300 ng/ml) or Pam3-CSK4 (TLR2; 300 ng/ml) or ODN (TLR9; 1 µg/ml). Subsequently, cells were harvested, followed by RNA isolation and qPCR analysis to quantify the levels of TNF-α, IL-6, and IFN-γ. **(D-E)** **Omp25 and its variants suppress LPS-induced production of pro-inflammatory cytokines in a dose-dependent manner.** RAW264.7 cells were transfected with various concentrations (100, 250, and 500 ng) of plasmid expressing MYC-Omp25 or its variants for 24 hours, followed by inducing the transfected cells with LPS (300 ng/ml) for 5 hours. The levels of TNF-α and IL-6 were quantified by qPCR. (**F-G) Omp25d overexpressed through lentivirus suppresses TLR-induced pro-inflammatory cytokines.** RAW264.7 cells were transduced with lentiviral particles harboring Omp25d or empty vector for 48 hours, followed by induction with LPS (300 ng/ml), Pam3-CSK4 (300 ng/ml) or ODN (1 μg/ml). Subsequently, cells were collected and levels of TNF-α and IL-6 were quantified by qPCR **(H) Recombinant MBP-Omp25 protein suppresses the expression of TNF-α in macrophages.** RAW264.7 cells were treated with MBP-Omp25d or MBP alone for 3 hours, followed by induction with various TLR ligands such as LPS, Pam3-CSK4 and ODN. Subsequently, the cells were harvested and the levels of TNF-α was quantified using qPCR
