# Supplementary material for: Outer membrane protein 25 of Brucella suppresses TLR-mediated expression of proinflammatory cytokines through degradation of TLRs and adaptor proteins
Source: J Biol Chem. 2023 Sep 29;299(11):105309. doi: 10.1016/j.jbc.2023.105309 (PMC10641269; doi:10.1016/j.jbc.2023.105309)
Supplement: Supporting Figure S3 [file mmc3.docx]

**
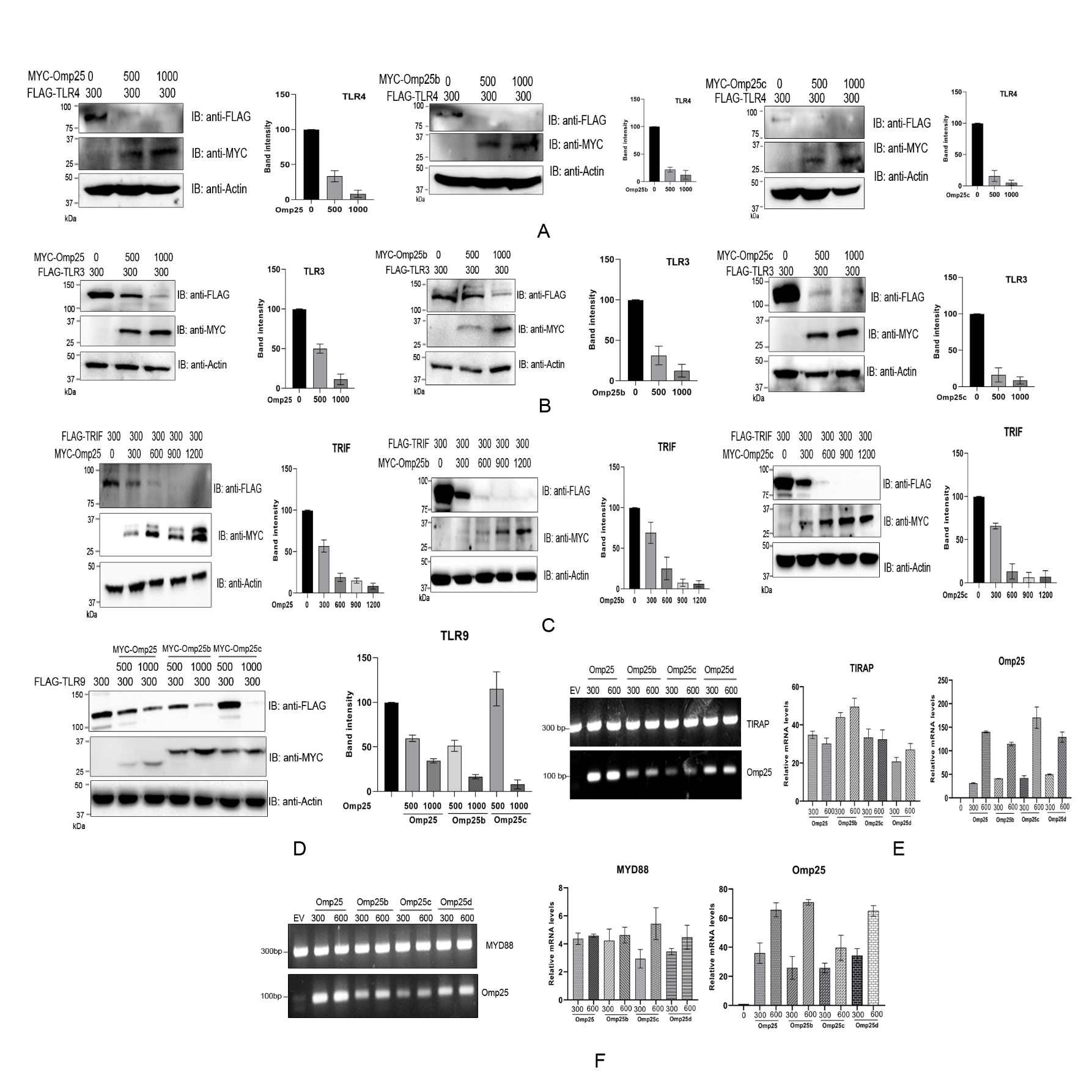
Supporting information Figure 3**

**Supporting information 3(A-D) Omp25 induces degradation of TLRs and their adapter proteins.** HEK293T cells were co-transfected with indicated concentrations of FLAG TLR4/ TLR3/ TRIF/ TLR9 and MYC/HA-tagged Omp25 or its variants. Cells were lysed 24 hours post-transfection, followed by immunoblotting. **(E-F) The transcript level of TIRAP or MYD88 in the presence of Omp25 and its variants**. HEK293T cells were transfected with FLAG-TIRAP (300 ng) and Omp25 or its variants (300 and 600 ng). Twenty-four hours after the transfections, total RNA was extracted from the cells, followed by cDNA synthesis and endpoint PCR or qPCR analysis. The transcript levels of TIRAP or MYD88 were not altered in the presence of Omp25 and its variants.
