# Supplementary material for: Outer membrane protein 25 of Brucella suppresses TLR-mediated expression of proinflammatory cytokines through degradation of TLRs and adaptor proteins
Source: J Biol Chem. 2023 Sep 29;299(11):105309. doi: 10.1016/j.jbc.2023.105309 (PMC10641269; doi:10.1016/j.jbc.2023.105309)
Supplement: Supporting Figure S4 [file mmc4.docx]

**
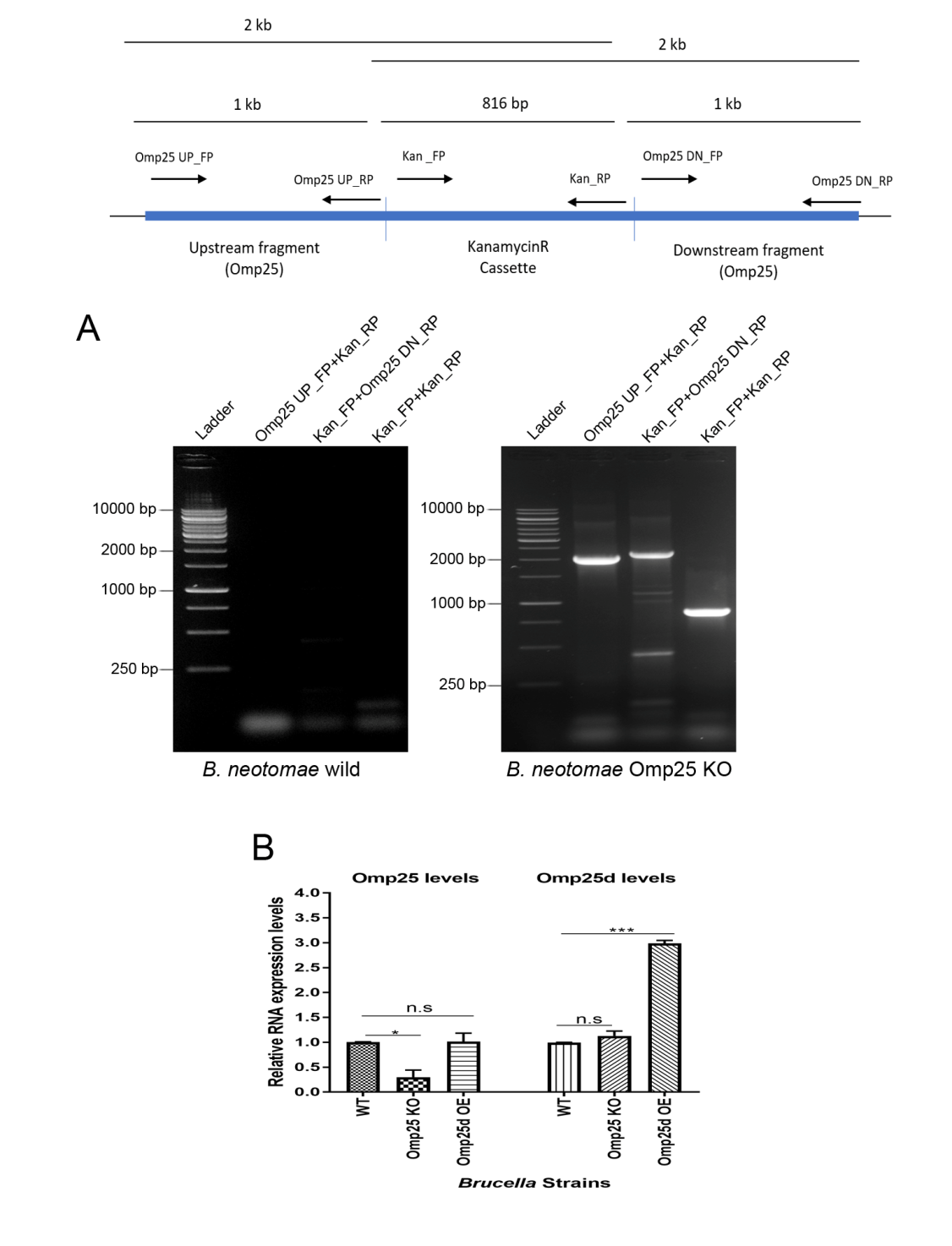
Supporting information Figure 4**

**Supporting information 4: (A) Confirmation of Omp25 KO *B. neotomae* by PCR.** WT or Omp25 KO *B. neotomae* was cultured until 1 O.D at 600 nm. Subsequently, the genomic DNA was isolated, followed by PCR using various primer combinations as indicated. **(B) Omp25 and Omp25d expression levels in WT/Omp25 KO/*B. neotomae* overexpressing Omp25d.** The indicated *B. neotomae* strains were cultured until 1 O.D at 600 nm. Subsequently, total RNA was isolated, followed by cDNA synthesis and qPCR analysis using Omp25 and Omp25d specific primers. All data were normalized with 16srRNA, and the relative expression was quantified with respect to the WT.
