# Supplementary material for: Outer membrane protein 25 of Brucella suppresses TLR-mediated expression of proinflammatory cytokines through degradation of TLRs and adaptor proteins
Source: J Biol Chem. 2023 Sep 29;299(11):105309. doi: 10.1016/j.jbc.2023.105309 (PMC10641269; doi:10.1016/j.jbc.2023.105309)
Supplement: Supporting Figure S5 [file mmc5.docx]

**
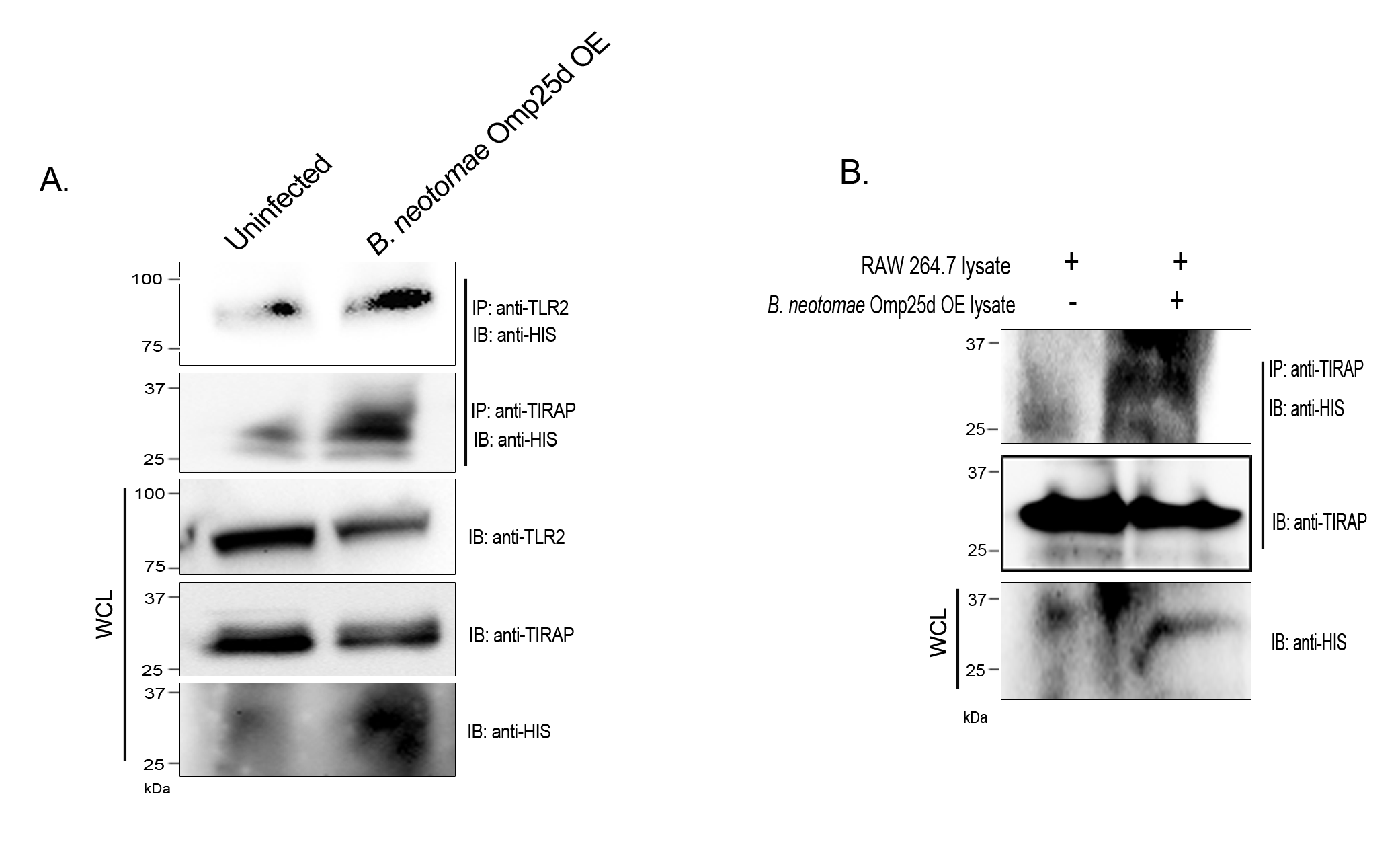
Supporting information Figure 5**

**Supporting information Figure 5: Omp25 interacts with TIRAP and TLR2 in the *Brucella-*infected macrophages. (A)** RAW264.7 cells were infected with *B. neotomae* overexpressing HIS-Omp25d, followed by the pull-down of HIS-Omp25d using Nickle-NTA resin and immunoblotting. The membranes were probed with anti-TIRAP or anti-TLR2 primary antibody, followed by HRP-conjugated anti-rabbit IgG to detect the respective proteins. Anti-HIS-HRP was used for detecting HIS-Omp25d**. (B) Co-immunoprecipitation of TIRAP and Omp25.** The lysates of RAW264.7 was incubated with *B. neotomae* overexpressing HIS-Omp25d culture lysate, followed by immunoprecipitation of TIRAP using anti-TIRAP antibody and immunoblotting. The membrane was probed with HRP- conjugated anti-HIS antibody to detect HIS-Omp25d.
