# Supplementary material for: Outer membrane protein 25 of Brucella suppresses TLR-mediated expression of proinflammatory cytokines through degradation of TLRs and adaptor proteins
Source: J Biol Chem. 2023 Sep 29;299(11):105309. doi: 10.1016/j.jbc.2023.105309 (PMC10641269; doi:10.1016/j.jbc.2023.105309)
Supplement: Supporting Figure S6 [file mmc6.docx]

**
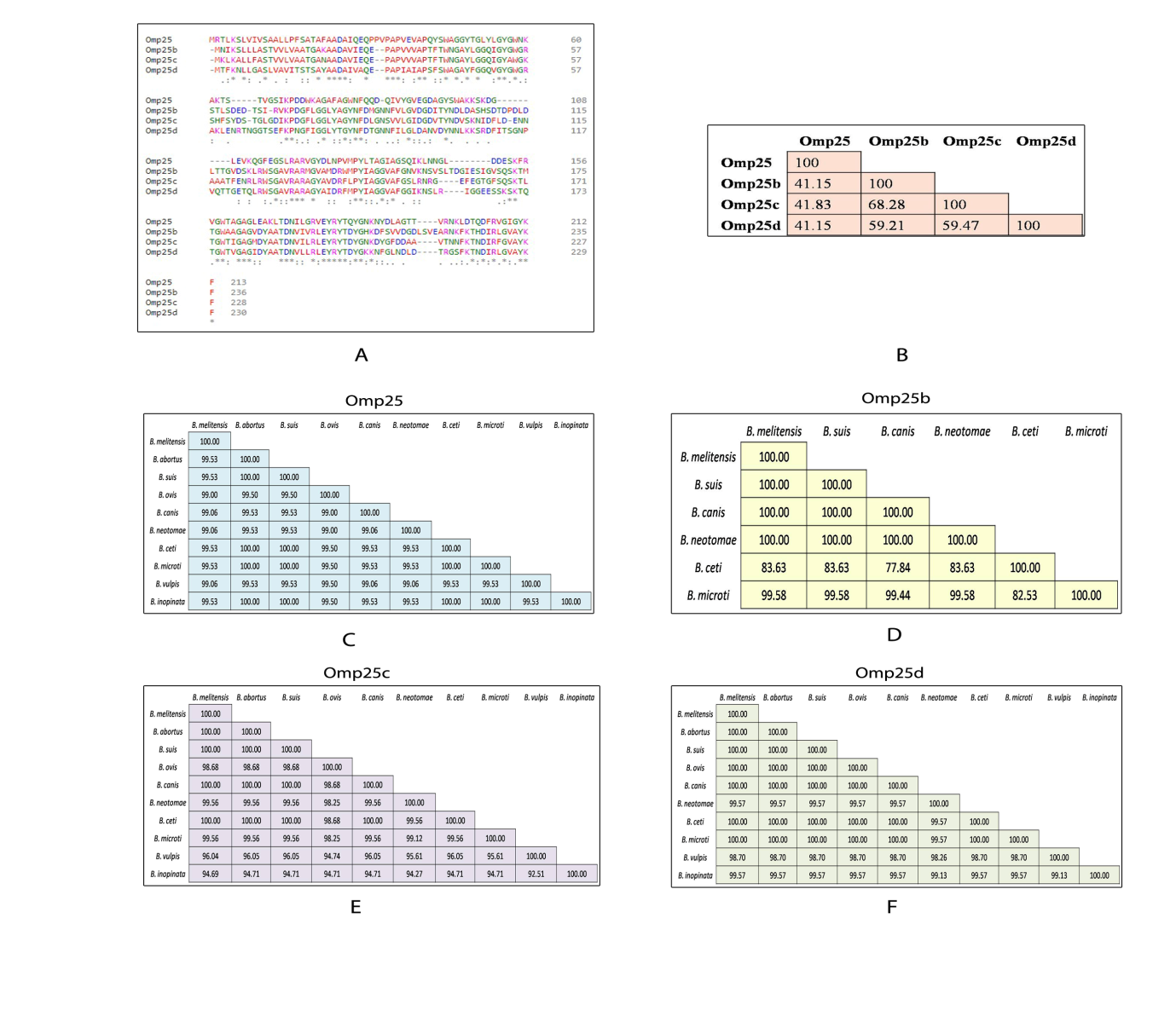
Supporting information Figure 6**

**Supporting information 6 (A)** Multiple sequence alignment of Omp25 and its variants from *B. melitensis*. The asterisk indicates the conserved amino acids among the variants **(B)** The percentage of identity between different variants of Omp25 from *B. melitensis.* **(C-F)** The inter-species percentage identity of Omp25 (C) Omp25b (D) Omp25c (E) Omp25d (F).
